# Impact of low dose inhaled nitric oxide treatment in spontaneously breathing and intubated COVID-19 patients: a retrospective propensity-matched study

**DOI:** 10.1186/s13054-024-05093-w

**Published:** 2024-10-25

**Authors:** Shahin Isha, Prasanth Balasubramanian, Abby J. Hanson, Sadhana Jonna, Lekhya Raavi, Subekshya Khadka, Ananya Vasudhar, Jorge Sinclair De Frias, Anna Jenkins, Arvind Balavenkataraman, Aysun Tekin, Vikas Bansal, Swetha Reddy, Sean M. Caples, Syed Anjum Khan, Nitesh K. Jain, Abigail T. LaNou, Rahul Kashyap, Rodrigo Cartin-Ceba, Ricardo Diaz Milian, Carla P. Venegas, Anna B. Shapiro, Anirban Bhattacharyya, Sanjay Chaudhary, Sean P. Kiley, Quintin J. Quinones, Neal M. Patel, Pramod K. Guru, Pablo Moreno Franco, Devang K. Sanghavi

**Affiliations:** 1 Department of Critical Care Medicine, Mayo Clinic, 4500 San Pablo Rd S, Jacksonville, FL 32224 USA; 2 Department of Pulmonary and Critical Care, Mayo Clinic, Jacksonville, FL USA; 3 Mayo Clinic Alix School of Medicine, Jacksonville, FL USA; 4 Department of Critical Care Medicine, Mayo Clinic, Rochester, MN USA; 5 Division of Pulmonary and Critical Care, Mayo Clinic, Rochester, MN USA; 6 Department of Critical Care Medicine, Mayo Clinic Health System, Mankato, MN USA; 7 Emergency Medicine and Critical Care, Mayo Clinic Health System, Eau Claire, WI USA; 8 Department of Anesthesia and Critical Care Medicine, Mayo Clinic, Rochester, MN USA; 9 Department of Critical Care Medicine, Mayo Clinic, Phoenix, AZ USA

**Keywords:** COVID-19, ARDS, HFNC, Inhaled nitric oxide, Non-invasive ventilation

## Abstract

**Background:**

The benefit of Inhaled nitric oxide (iNO) therapy in the setting of COVID-19-related ARDS is obscure. We performed a multicenter retrospective study to evaluate the impact of iNO on patients with COVID-19 who require respiratory support.

**Methods:**

This retrospective multicenter study included COVID-19 patients enrolled in the SCCM VIRUS COVID-19 registry who were admitted to different Mayo Clinic sites between March 2020 and June 2022 and required high-flow nasal cannula (HFNC), non-invasive ventilation (NIV), or invasive mechanical ventilation (IMV). Patients were included in the ‘spontaneously breathing’ group if they remained non-intubated or were initiated on an HFNC (± NIV) before intubation. Patients who got intubated without prior use of an HFNC (± NIV) were included in the ‘intubated group.’ They were further divided into categories based on their iNO usage. Propensity score matching (PSM) and inverse propensity of treatment weighting (IPTW) were performed to examine outcomes.

**Results:**

Among 2767 patients included in our analysis, 1879 belonged to spontaneously breathing (153 received iNO), and 888 belonged to the intubated group (193 received iNO). There was a consistent improvement in FiO2 requirement, P/F ratio, and respiratory rate within 48 h of iNO use among both spontaneously breathing and intubated groups. However, there was no significant difference in intubation risk with iNO use among spontaneously breathing patients (PSM OR 1.08, CI 0.71–1.65; IPTW OR 1.10, CI 0.90–1.33). In a time-to-event analysis using Cox proportional hazard model, spontaneously breathing patients initiated on iNO had a lower hazard ratio of in-hospital mortality (PSM HR 0.49, CI 0.32–0.75, IPTW HR 0.40, 95% CI 0.26–0.62) but intubated patients did not (PSM HR: 0.90; CI 0.66–1.24, IPTW HR 0.98, 95% CI 0.73–1.31). iNO use was associated with longer in-hospital stays, ICU stays, ventilation duration, and a higher incidence of creatinine rise.

**Conclusions:**

This retrospective propensity-score matched study showed that spontaneously breathing COVID-19 patients on HFNC/ NIV support had a decreased in-hospital mortality risk with iNO use in a time-to-event analysis. Both intubated and spontaneously breathing patients had improvement in oxygenation parameters with iNO therapy but were associated with longer in-hospital stays, ICU stays, ventilation duration, and higher incidence of creatinine rise.

**Supplementary Information:**

The online version contains supplementary material available at 10.1186/s13054-024-05093-w.

## Background

Acute Respiratory Distress Syndrome (ARDS) is characterized by acute hypoxemic respiratory failure with onset less than seven days, worsening respiratory symptoms, and bilateral opacities that are not explained by effusions, lung collapse, or nodules as seen on chest imaging. It develops in the background of a known clinical insult to the lung in the absence of any cardiogenic cause [1–3]. The presence of alveolar inflammation and the resulting accumulation of fluid rich in proteins and alveolar inflammatory cells leads to reduced lung compliance, ventilation-perfusion mismatch, and subsequent development of hypoxia.

Traditionally, the management of ARDS is focused on resolving the underlying cause as well as providing supportive treatment. In cases of severe refractory hypoxia, treatment with inhaled nitric oxide (iNO) can be employed. Inhaled nitric oxide has the potential for preferential pulmonary vasodilation in the ventilated lung parenchyma, improving ventilation-perfusion mismatch and thereby enhancing arterial oxygenation [4, 5]. Additionally, iNO has been shown to induce bronchodilation and decrease vascular endothelium inflammation platelet aggregation, thereby reducing intra-pulmonary microthrombosis [6, 7].

The use of iNO in adult ARDS patients stems back to 1993 when improvements in PaO2/FiO2 ratio and reduction of pulmonary artery pressure were observed in severe ARDS, as reported by Roissant et al. [4]. Subsequent literature reported that despite a role in improving oxygenation, benefits with iNO were found to be limited in terms of overall hospitalization outcomes [8]. A systematic review with meta-analysis comprising 13 clinical trials of 1243 patients showed there were no significant differences in ventilator-free days, duration of mechanical ventilation, or length of stay in the intensive care unit or hospital, although a significant improvement in oxygenation index was noted at 24 h of iNO therapy [9]. Similar findings were reported in a previous systematic review conducted by Adhikary et al. [10]. Both studies reported an association between renal impairment and iNO therapy. Given the lack of significant survival benefits, there is a paucity of clinical evidence supporting the routine use of iNO in the ARDS population, but it is often considered in patients who remain severely hypoxemic despite optimal ventilation and rescue strategies [11, 12].

The Coronavirus Disease 2019 (COVID-19) pandemic has posed a global challenge with its varying levels of disease severity and clinical manifestations. Wu et al. (2020) reported that COVID-19-associated ARDS (CARDS) can develop in up to 42% of patients, with 61% to 81% of them requiring treatment in intensive care units [13]. A significant portion of CARDS patients require mechanical ventilation due to hypoxemic respiratory failure, and the estimated mortality rates range between 26 and 88% [14–18]. Despite the lack of clinical guidelines on the use of iNO in COVID-19 ARDS, studies have reported the use of iNO in an attempt to improve oxygenation, often in refractory hypoxemia [19–22]. Moreover, during the SARS-CoV-1 epidemic in 2004, it was also hypothesized that iNO might have antiviral properties; therefore, COVID-19 patients may also demonstrate similar benefits [23]. A multicenter, randomized, placebo-controlled trial performed on 385 patients with moderate to severe lung injury demonstrated that low-dose iNO led to short-term improvement in oxygenation status but did not decrease the duration of ventilatory support or mortality [24]. The literature afterward reported conflicting results, with some showing improvement in oxygenation indices and some not showing benefit from therapy [25–29]. A recent study conducted by Chandel et al. also reported the findings associated with iNO use in spontaneously breathing, non-intubated COVID-19 patients. They found that iNO delivered via high-flow nasal cannula (HFNC) did not reduce oxygen requirements in the majority of patients or improve clinical outcomes [26]. Di Fenza et al., in their phase II multicenter trial, also found that the use of high-dose inhaled nitric oxide resulted in improvement in PaO2/FiO2 ratio at 48 h compared to the usual care group among intubated COVID-19 patients, although they did not notice any mortality difference [30]. A recently published systematic review and meta-analysis comprising 17 studies on this topic pointed out the variability in study findings and limitations associated with small sample sizes, the median study sample being 34 patients [31].

On the background of limited evidence on spontaneously and non-spontaneously breathing COVID-19 patients, we conducted a multicenter retrospective study, including hospitalized COVID-19 ARDS patients who were supported on invasive ventilation, non-invasive ventilation, or high flow nasal cannula and compared their oxygenation indices as well as hospital outcomes based on presence or absence of iNO therapy.

## Methods

### Study design

This multicenter, retrospective cohort study includes all critically ill hospitalized COVID-19 patients who developed hypoxic respiratory failure and were supported by either HFNC, Non-invasive ventilation (BiPAP), or mechanical ventilation. Patients were included in the ‘spontaneously breathing’ group if they were put on an HFNC (± NIV) before intubation or received HFNC (± NIV) treatment without getting intubated. Patients included in the ‘intubation group’ were those who got intubated without prior use of an HFNC/NIV. Patients were divided into two groups based on whether they received iNO during their hospitalization. Patients who received iNO therapy and also were intubated were further subdivided into two groups based on the timing of iNO initiation: pre-intubation (those who were started on iNO while on HFNC/ NIV support before intubation) and post-intubation (those who were started on iNO after intubation as a measure to improve oxygenation or treat refractory hypoxemia). All patients were followed until the day they were discharged from the hospital or died.

### Ethics

This study was performed under the exemption criteria from the Mayo Clinic Institutional Review Board. Access to the multicenter Mayo Clinic data was granted through “Viral Infection and Respiratory Illness Universal Study [VIRUS]: COVID-19 Registry and Validation of C2D2 (Critical Care Data Dictionary)” under IRB ID 20–002610. The need for informed consent was waived by the IRB due to its retrospective design, data anonymity, and non-interventional nature.

### Study population

**Fig. 1:**
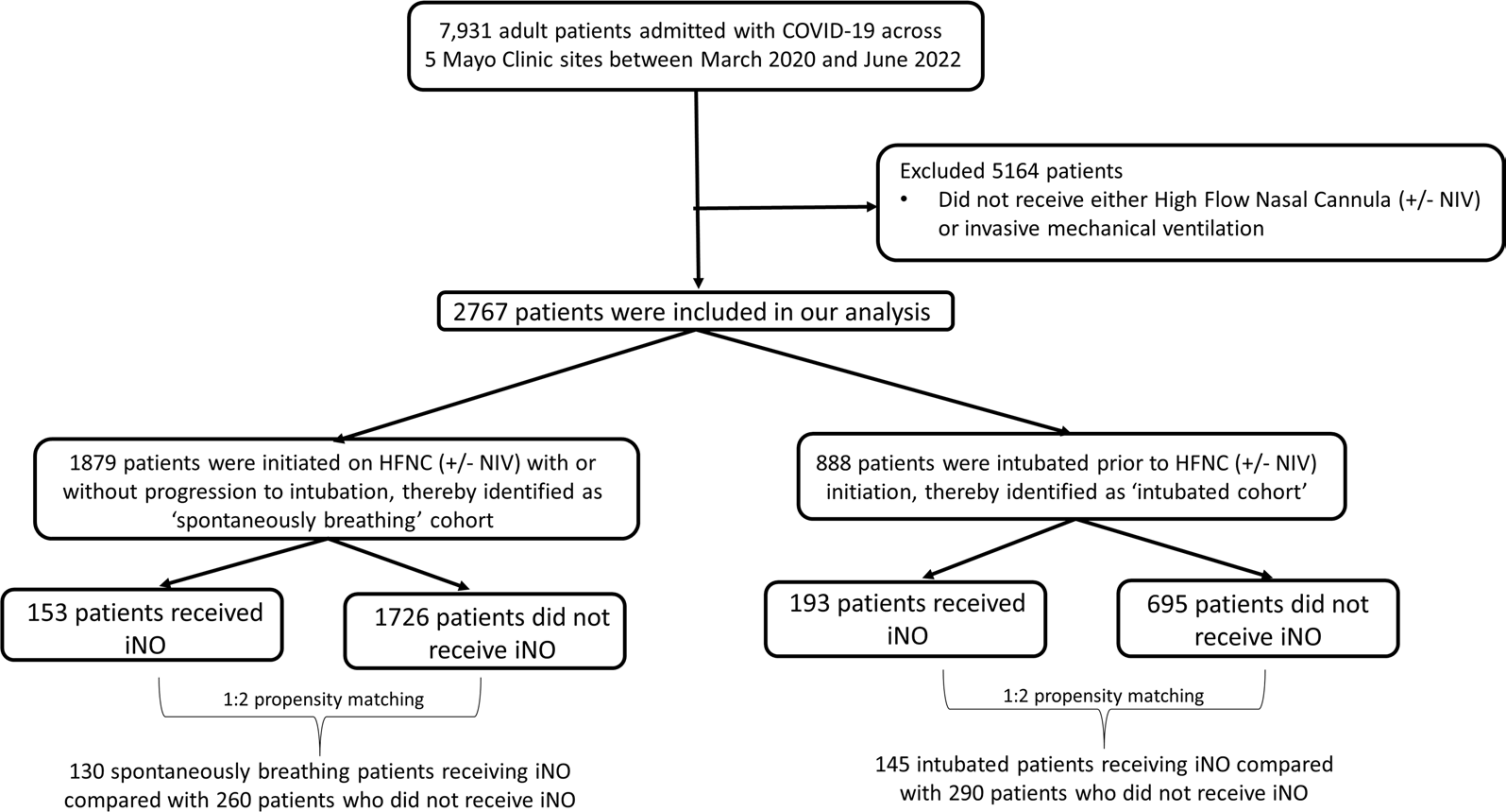
A CONSORT diagram depicting the inclusion/exclusion criteria for our study cohort

This study included adult COVID-19 patients who were admitted to different Mayo Clinic sites between March 2020 and June 2022 and were enrolled in the Society of Critical Care Medicine VIRUS COVID-19 registry [32–34]. Patients were included in the analysis if they developed hypoxic respiratory failure and needed respiratory support by HFNC, NIV, or mechanical ventilation. Patients were excluded if they were aged less than 18 years, lacked research authorization, or did not receive ventilatory support by high-flow nasal cannula or invasive mechanical ventilation. Patients who received only BiPAP but not HFNC were not included in the “spontaneously breathing” group as the indication of BiPAP could not be confirmed (whether hypoxic respiratory failure or home usage for obstructive sleep apnea). Figure 1 demonstrates the CONSORT diagram for our study design.

### Inhaled nitric oxide setup

On the background of limited yet conflicting data on the use of iNO in COVID-19 patients, its application in the Mayo Clinic System was semi-protocolized [35]. Among hypoxic patients who sustained desaturation (SpO2 < 85% and/or PaO2 < 60) despite being on reservoir NC or NRBM, high flow nasal cannula (60 LPM, 50–80% FiO2) were often initiated if work of breathing was relatively low. A trial of 5–20 ppm iNO was often added to the HFNC if patients were having pure hypoxemia without increased work of breathing (or respiratory acidosis). The presence of pulmonary hypertension or RV failure were other factors favoring the use of iNO in such situations. Most importantly, clinician judgment and provider preference were significant in such scenarios. A similar strategy was adopted in mechanically ventilated patients demonstrating refractory hypoxia [35].

iNO is delivered to the patient through a non-invasive method such as HFNC, a non-invasive positive airway pressure machine, or an invasive mechanical ventilator. The inhaled nitric oxide via the INOmax DSIR Plus machine is connected to the usual care setup of oxygen therapy.

### HFNC

High-pressure oxygen and air at 40 psi from the hospital wall supply are y-sited into a blender to provide a prescribed FiO2. The prescribed FiO2 travels through a flowmeter at the provider’s prescription, ranging from between 10 to 60 L/min. Just before the oxygen and air combination reaches the humidifier, it is connected to an injection tubing that comes from the iNO machine. The machine delivers a prescribed amount of iNO. All of these gases then travel to a heater/humidifier at 31 degrees Celsius and 100% humidity. From here, the gas combination travels through tubing on its way to reach the patient's HFNC prongs. Before the mixture reaches the patient, sampling-tubing is t-pieced to measure the amount of iNO (ppm) that reaches the patient. The machine reads this amount and adjusts the amount of iNO that is delivered to the patient through the injection tubing at the beginning, as some is lost before it reaches the patient. In the end, the prescribed FiO2, liters of oxygen, and the calculated ppm of iNO travel through tubing to the patient at the prescribed doses.

### BiPAP/ETT

High-pressure oxygen and air at 40 psi are y-sighted to the ventilator. The ventilator combines this air to allow the correct FiO2 and other provider-prescribed settings to reach the patient. From the machine, the air travels to an injector where iNO, the nitric oxide machine, enters the mix of air, oxygen, and gas. This is all transferred to a heater/humidifier at 31 degrees Celsius and 100% humidity. This mixture travels to the patient. Just before it reaches the ETT (invasive ventilation) or mask (NIV such as BiPAP/CPAP), a sampling tubing comes from the iNO machine and is in place. It measures the concentration of iNO that is actually reaching the patient and adjusts the amount that the patient receives through the injector tubing as some iNO is lost during the humidification process.

### Data collection

Data were collected by retrospective chart review of patients in our cohort who received iNO therapy. Manual data extraction was performed on RedCap interface to collect the initiation dose and timing of nitric oxide therapy, oxygenation indices at admission, and different intervals after iNO therapy. Automated data pull collected information related to patient demographics, hospital outcomes, timing and duration of non-invasive and invasive ventilation, and baseline severity scores such as SOFA score. All data were collected and handled while maintaining anonymity and confidentiality and were stored in a secure intranet database.

### Outcomes

Our primary endpoint was assessing the improvement in oxygenation indices (P/F ratio, FiO2 requirement, and respiratory rate). The secondary outcomes of interest were assessing the risk of intubation, in-hospital mortality, creatinine rise, continuous renal replacement therapy (CRRT) requirement, extracorporeal membrane oxygenation (ECMO) length of invasive mechanical ventilation, hospital length of stay, and ICU length of stay among patients with iNO in patients on high-flow nasal cannula. ‘Creatinine rise’ was defined per KDIGO criteria as an increase in creatinine 0.3 mg/dL within 48 h or an increase in creatinine ≥ 1.5 times baseline during the hospital stay[36]. Due to the lack of accurate hourly urine output data in our database, we were unable to identify who fulfilled (or did not fulfill) the complete KDIGO criteria for AKI. We also performed additional subgroup analysis wherein patients identified as “responders” were compared with “non-responders.” Patients were defined as responders if they had improved oxygenation status (decreased FiO2 requirement and/ or increased P/F ratio) within 48 h of iNO initiation. For mechanically ventilated patients, vent-free days (VFD) were calculated as the number of days spent on mechanical ventilation deducted from 28 days (28-total number of days spent on mechanical ventilation). Any patient who died within 28 days of mechanical ventilation initiation or had more than 28 days spent on invasive ventilation was assigned zero (0) vent-free days. Similarly, ICU-free days were calculated as the number of days spent in ICU deducted from 28 days (28-total number of days spent in ICU). Any patient who died within 28 days of hospital admission or had more than 28 days spent in ICU was assigned zero (0) ICU-free days.

### Statistical analysis

Data analyses were conducted using BlueSky software version 10.3.1 and R Studio, with R version 4.2.1. We presented categorical data as percentages and assessed differences using the Chi-square test. Median values, along with the first and third quartiles, represented continuous data, and differences between groups were evaluated using the Wilcoxon rank sum test for independent samples, with a significance threshold set at a p-value less than 0.05. For patients treated with inhaled nitric oxide, oxygenation indices before and after iNO treatment were compared using Wilcoxon paired sample tests. All analyses were performed separately for spontaneously breathing and post-intubation group patients.

For propensity score modeling, variables identified as relevant were included using the "MatchIt" package in R [37]. Patients were excluded if they had key variables missing that would deny us from generating propensity scores. Among 958 spontaneously breathing patients (137 iNO group and 821 non-iNO), who did not have any key variables missing, the propensity score was calculated for each individual through logistic regression. The ‘genetic matching’ method served as the matching algorithm, employing the Generalized Linear Model (glm) as the distance metric, with a preset caliper distance of 0.2. Matching was conducted on a 1:2 basis without replacement (130 patients who received iNO treatment were matched with 260 similar patients in the non-iNO group). We then evaluated the balance of covariates post-matching to confirm the effectiveness of the matching process. We considered a standardized median difference below 0.1 indicative of a minor imbalance and above 0.2 as a significant imbalance [38]. Variables included in the propensity matching method, as well as respective covariate balances, are demonstrated using the Supplementary file 1: Figure S1 (built using the "love.plot" package in R). Subsequently, we applied a univariate logistic regression model to the matched data, incorporating "weights" based on the propensity scores, to investigate the causal association between the outcomes of interest (creatinine rise, intubation, CRRT requirement) and iNO use. Linear regression models were adopted for non-binary outcomes (IMV length, ICU LOS, Hospital LOS). Survival analysis was performed using the Cox univariate regression model, with the day since HFNC start (for spontaneously breathing patients) or the time since intubation (for the post-intubation group) as the time variable [39]. A similar methodology was adopted to match 145 patients who received iNO after intubation with 290 patients who did not receive iNO after intubation.

Anticipating the impact of reduced sample size due to 1:2 matching, we also applied inverse propensity of treatment weighting (IPTW) based on propensity scores derived from a separate logistic regression that included all variables used in propensity matching. Similar to propensity matching, patients were excluded if they had key variables missing. 958 spontaneously breathing patients (137 in iNO group and 821 non-iNO), who did not have any key variables missing, were therefore included in the IPTW model. The IPTW formula used was: IPTW = [(iNO use/Propensity) + ((1–iNO use)/(1—Propensity))]. We created a distinct dataset of 958 patients, each assigned an IPTW. We then conducted separate regression analyses on this dataset, using "weights" defined as the inverse of the propensity weight of treatment (ipw), to explore the association between iNO use and outcomes of interest. A similar methodology was adopted on 666 intubated patients without missing key variables (171 received iNO and 495 did not receive iNO), to explore the effect of post-intubation iNO usage on our primary and secondary outcomes of interest. Figure 1

## Results

During the study period, 8098 patients were screened. Of these patients, 346 received inhaled nitric oxide (iNO). We subdivided our patients into two cohorts: those who were spontaneously breathing with (n = 153) or without iNO (n = 1726) use and those who were intubated then were started (n = 193) or were not started (n = 695) on iNO.

### *Demographics and patient characteristics: (*Tables 1, 2*)*

**Table 1:** Spontaneously breathing patients who were started on HFNC/ NIV with or without iNO use

Variables	Before matching			After matching		
	No iNO used (n = 1726)	iNO used (n = 153)	SMD	No iNO used (n = 260)	iNO used (n = 130)	SMD
Age	63.37 (16.58)	62.03 (13.47)	0.089	62.23 (14.31)	62.35 (13.65)	0.008
Female Sex	613 (35.5%)	52 (34.0%)	0.032	76 (29.2%)	43 (33.1%)	0.083
Race			**0.308**			0.084
Caucasian	1460 (84.6%)	119 (77.8%)		213 (81.9%)	103 (79.2%)	
American Indian/Alaska Native/ Pacific Islander	46 (2.7%)	5 (3.3%)		7 (2.7%)	5 (3.8%)	
African American	43 (2.5%)	12 (7.8%)		12 (4.6%)	7 (5.4%)	
Asian	68 (3.9%)	11(7.2%)		16 (6.2%)	9 (6.9%)	
Mixed/Other/Unknown	109 (6.3%)	6 (3.9%)		12 (4.6%)	6 (4.6%)	
BMI	32.07 (8.12)	31.30 (6.07)	0.108	31.23 (5.81%)	31.36 (6.37%)	0.022
Highest SOFA	7.02 (4.03)	9.02 (4.24)	**0.485**	8.68 (4.33)	8.68 (4.07)	< 0.001
Baseline SOFA	4.59 (2.36)	5.12 (2.08)	**0.236**	5.34 (2.58)	4.98 (1.99)	0.157
CAD	155 (9.0%)	15 (9.8%)	0.028	23 (8.8%)	14 (10.8%)	0.065
HTN	672 (38.9%)	66 (43.1%)	0.086	108 (41.5%)	54 (41.5%)	< 0.001
CHF	45 (2.6%)	1 (0.7%)	0.155	2 (0.8%)	1 (0.8%)	< 0.001
CKD	146 (8.5%)	11 (7.2%)	0.047	15 (5.8%)	8 (6.2%)	0.016
DM	412 (23.9%)	37 (24.2%)	0.007	64 (24.6%)	31 (23.8%)	0.018
HLD	517 (30.0%)	55 (35.9%)	0.128	84 (32.3%)	46 (35.4%)	0.065
Pre-hospital obesity diagnosis	158 (9.2%)	16 (10.5%)	0.044	23 (8.8%)	15 (11.5%)	0.089
COVID-19 vaccination	802 (46.5%)	64 (41.8%)	0.093	116 (44.6%)	56 (43.1%)	0.031
Baseline code status			**0.252**			0.125
Full code	1486 (86.1%)	143 (93.5%)		246 (94.6)	122 (93.8%)	
No CPR, Yes intubation	20 (1.2%)	1 (0.7%)		0 (0.0%)	1 (0.8%)	
Yes CPR, No intubation	22 (1.3%)	1 (0.7%)		2 (0.8%)	1 (0.8%)	
DNR/DNI	192 (11.1%)	8 (5.2%)		12 (4.6%)	6 (4.6%)	
Unknown	6 (0.3%)	0 (0.0%)		0 (0.0%)	0 (0.0%)	
Creatinine rise	780 (45.2%)	115 (75.2%)	**0.643**	168 (64.6%)	99 (76.2%)	**0.255**
IMV	301 (17.4%)	68 (44.4%)	**0.611**	109 (41.9%)	57 (43.8%)	0.039
ECMO	7 (0.4%)	13 (8.5%)	**0.400**	1 (0.4%)	8 (6.2%)	**0.329**
CRRT	53 (3.1%)	16 (10.5%)	**0.297**	20 (7.7%)	12 (9.2%)	0.055
HFNC duration (mean)	4.44 (4.48)	11.03 (11.95)	**0.731**	5.03 (5.67)	11.28 (11.64)	**0.683**
NIMV Days (mean)	2.24 (2.45)	2.34 (2.61)	0.036	1.67 (1.75)	2.42 (2.70)	**0.327**
IMV Days (mean)	9.40 (8.28)	15.48 (15.09)	**0.499**	10.13 (8.09)	14.85 (13.56)	**0.424**
Vent-free days	12.18 (11.15)	9.18 (10.69)	**0.275**	11.82 (10.75)	8.36 (9.62)	**0.340**
ECMO duration hospitalization (mean)	16.43 (18.24)	38.62 (24.98)	**1.015**	7.00 (N/A)	40.88 (24.98)	**NA**
CRRT duration (mean)	8.79 (8.77)	10.31 (10.44)	0.158	9.30 (9.56)	9.92 (9.43)	0.065
ICU LOS (mean)	7.65 (7.69)	18.82 (19.42)	**0.756**	10.36 (8.95)	18.97 (18.02)	**0.605**
ICU-free days	20.62 (6.66)	13.23 (9.68)	**0.890**	18.07 (7.73)	12.67 (9.56)	**0.621**
Hospital LOS (mean)	12.61 (9.35)	26.05 (23.96)	**0.739**	16.92 (12.76)	25.91 (20.52)	**0.526**
In-hospital mortality	278 (16.1%)	36 (23.5%)	0.187	63 (24.2%)	29 (22.3%)	0.046

SMD > 0.2 signifies significant difference between the groups

CAD: Coronary artery disease; HTN: hypertension; CHF: congestive heart failure; CKD: chronic kidney disease; BMI: body mass index, SOFA: sequential organ failure assessment; IMV: invasive mechanical ventilation; NIV: non-invasive ventilation; HFNC: high-flow nasal cannula; ECMO: extracorporeal membrane oxygenation; CRRT: continuous renal replacement therapy; LOS: length of stay. Statistically significant p-values (<0.05) are highlighted in bold font.

**Table 2:** Intubated patients stratified by use of iNO

Variables	Before matching			After matching		
	No iNO (n = 695)	iNO used (n = 193)	SMD	No iNO (n = 290)	iNO used (n = 145)	SMD
Age (mean)	60.21 (16.45)	57.27 (14.20)	0.191	59.20 (14.70)	58.58 (14.40)	0.043
Female Sex	236 (34.0%)	65 (33.7%)	0.006	99 (34.1%)	54 (37.2%)	0.065
Race			**0.342**			0.136
Caucasian	554 (79.7%)	140 (72.5%)		218 (75.2%)	105 (72.4%)	
American Indian/Alaska Native/Pacific Islander	18 (2.6%)	17 (8.8%)		13 (4.5%)	11 (7.6%)	
African American	15 (2.2%)	10 (5.2%)		11 (3.8%)	6 (4.1%)	
Asian	24 (3.5%)	9 (4.7%)		15 (5.2%)	8 (5.5%)	
Mixed/other/unknown	84 (12.1%)	17 (8.8%)		33 (11.4%)	15 (10.3%)	
BMI (mean)	32.95 (8.60)	32.83 (7.64)	0.014	32.36 (7.33)	32.92 (8.11)	0.072
Highest SOFA	12.24 (2.92)	13.83 (2.54)	**0.580**	13.34 (2.40)	13.39 (2.40)	0.020
CAD	54 (7.8%)	13 (6.7%)	0.040	22 (7.6%)	12 (8.3%)	0.026
HTN	233 (33.5%)	66 (34.2%)	0.014	105 (36.2%)	53 (36.6%)	0.007
CHF	14 (2.0%)	2 (1.0%)	0.080	2 (0.7%)	2 (1.4%)	0.068
CKD	51 (7.3%)	14 (7.3%)	0.003	18 (6.2%)	12 (8.3%)	0.547
DM	138 (19.9%)	36 (18.7%)	0.031	63 (21.7%)	31 (21.4%)	0.008
HLD	151 (21.7%)	38 (19.7%)	0.050	70 (24.1%)	34 (23.4%)	0.016
Pre-hospital obesity diagnosis	56 (8.1%)	23 (11.9%)	0.129	19 (6.6%)	10 (6.9%)	0.014
COVID vaccination	275 (39.9%)	63 (32.6%)	0.145	109 (37.6%)	55 (37.9%)	0.007
Baseline code status			0.192			0.070
Full code	663 (95.4%)	188 (97.4%)		283 (97.6%)	140 (96.6%)	
No CPR, Yes Intubation	12 (1.7%)	4 (2.1%)		5 (1.7%)	4 (2.8%)	
Yes CPR, No intubation	4 (0.6%)	0 (0.0%)		0 (0.0%)	0 (0.0%)	
DNR/DNI	13 (1.9%)	1 (0.5%)		2 (0.7%)	1 (0.7%)	
Unknown	3 (0.4%)	0 (0.0%)		-	-	
Creatinine rise	534 (76.8%)	182 (94.3%)	**0.513**	251 (86.6%)	138 (95.2%)	**0.303**
ECMO	31 (4.5%)	51 (26.4%)	**0.638**	20 (6.9%)	34 (23.4%)	**0.474**
CRRT	92 (13.2%)	65 (33.7%)	**0.497**	62 (21.4%)	40 (27.6%)	0.145
NIMV Days (mean)	2.14 (2.91)	1.69 (2.19)	0.173	1.58 (2.32)	1.22 (1.41)	0.185
HFNC Duration (mean)	3.53 (3.92)	5.08 (7.23)	**0.265**	3.51 (3.66)	5.03 (7.48)	**0.259**
IMV Days (mean)	9.66 (11.92)	23.25 (23.40)	**0.732**	11.47 (12.49)	22.35 (19.30)	**0.670**
VFD at 28 days	13.50 (11.55)	5.44 (8.92)	**0.781**	11.98 (11.05)	5.85 (9.13)	**0.604**
ECMO Duration (mean)	37.16 (25.41)	43.73 (30.40)	**0.234**	35.30 (20.86)	41.56 (31.87)	**0.232**
CRRT duration (mean)	9.86 (10.32)	15.40 (15.55)	**0.497**	10.69 (11.41)	14.90 (13.95)	**0.330**
ICU LOS Days (mean)	13.19 (13.61)	28.12 (27.50)	**0.688**	15.27 (14.67)	27.59 (24.54)	**0.610**
ICUFD at 28 days	11.37 (10.37)	4.20 (7.46)	**0.794**	10.60 (10.25)	4.87 (8.16)	**0.619**
Hospital LOS Days(mean)	20.21 (16.93)	35.36 (33.16)	**0.576**	22.48 (17.26)	35.43 (31.87)	**0.505**
In hospital mortality	238 (34.2%)	98 (50.8%)	**0.339**	100 (34.5%)	65 (44.8%)	**0.213**

SMD > 0.2 signifies a significant difference between the groups

CAD: Coronary Artery Disease; HTN: Hypertension; CHF: Congestive Heart Failure; CKD: Chronic Kidney Disease; BMI: Body Mass Index, SOFA: Sequential Organ Failure Assessment; IMV: Invasive Mechanical Ventilation; NIV: Non-Invasive Ventilation; HFNC: High-Flow Nasal Cannula; ECMO: Extracorporeal Membrane Oxygenation; CRRT: Continuous Renal Replacement Therapy; LOS: Length of Stay. Statistically significant p-values (<0.05) are highlighted in bold font.

#### Spontaneously breathing patients (on HFNC/ NIV)

This cohort included 1879 spontaneously breathing patients. Among these, 153 received iNO while on HFNC/ NIV, and 1726 did not. Before propensity matching analysis, there was no significant difference in the distribution of age, sex, BMI, comorbidities, or COVID-19 vaccination rate. However, patients who received iNO were less likely to be Caucasian and have a higher baseline and maximum SOFA score. After a propensity score matching (1:2 ratio), we included 390 patients based upon predefined criteria; there was no significant imbalance in the distribution of demographics and comorbidities (all SMD < 0.2) between the two groups.

### Mechanically ventilated patients who were initiated on iNO after intubation

This cohort included 888 intubated patients. Of which, 193 were given iNO after intubation and 695 were not. Before propensity matching, there was no significant difference in demographics such as age, sex, BMI, COVID-19 vaccination or comorbidities. However, patients in iNO group were less likely to be Caucasians and had a higher maximum SOFA score. After a propensity score matching (1:2 ratio), we included 435 patients based on predefined criteria. There was no significant imbalance in the distribution of demographics and comorbidities (all SMD < 0.2) between the two groups.

### *Comparison of oxygenation and respiratory indices: (*Table 3*)*

**Table 3:** Comparison of oxygenation and respiratory indices across time in different patient subgroups

	At iNO initiation	24 h after iNO initiation	48 h after iNO initiation	*p* value (initiation vs 24 h)	*p* value (initiation vs 48 h)
Non-intubated					
Resp rate	24.0 (20.8, 28.0)	22.0 (19.0, 28.0)	22.0 (18.0, 25.5)	0.080	**0.002**
FiO2 req	100 (82.5, 100)	80.0 (68.7, 100.0)	70.0 (50.0, 100.0)	** < 0.001**	** < 0.001**
P/F ratio	68.9 (60.2, 84.0)	83.9 (72.5, 106.7)	76.9 (67.9, 95.1)	**0.039**	0.063
Progressed to intubation (> 48 h)					
Resp rate	22.0 (20.0, 27.0)	21.0 (18.0, 27.0)	22.0 (19.0, 26.0)	0.423	0.495
FiO2 req	100 (80.0, 100.0)	100.0 (80.0, 100.0)	100.0 (76.2, 100.0)	0.951	0.914
P/F ratio	61.9 (55.9, 72.1)	110.3 (77.6, 175.2)	124.8 (84.9, 148.9)	0.625	0.875
Post intubation					
Resp rate	24.0 (19.0, 28.0)	22.0 (15.0, 26.0)	20.0 (15.0, 26.0)	**0.003**	** < 0.001**
FiO2 req	100 (70.0, 100.0)	65.0 (50.0, 80.0)	50.0 (40.0, 75.0)	** < 0.001**	** < 0.001**
P/F ratio	80.0 (64.3, 113.3)	117.5 (88.7, 186.0)	127.5 (88.7, 187.0)	** < 0.001**	** < 0.001**
PEEP	12.0 (10.0, 14.0)	10.0 (10.0, 13.0)	10.0 (10.0, 14.0)	**0.007**	**0.025**

*P* < 0.05 denotes statistical significance. Pairwise comparisons were performed using the Wilcoxon paired sample test. FiO2: Fraction of inspired oxygen; P/F: PaO2: FiO2 ratio; PEEP: Positive end-expiratory pressure. Statistically significant p-values (<0.05) are highlighted in bold font.

#### Spontaneously breathing patients (on HFNC/ NIV)

We also analyzed oxygen indices and support requirements at initiation, 24 h post initiation, and 48 h post-initiation for patients who received iNO. For non-intubated patients who received iNO, there was a statistically significant difference in FiO2 requirements both at the 24- and 48-h post-initiation marks. There was also a statistically significant increase in the P/F ratio at 24 h post iNO initiation but not at 48 h. On the other hand, there was a significant decrease in respiratory rate after 48 h of iNO initiation, although such a difference was not apparent at 24 h.

### Mechanically ventilated patients who were initiated on iNO after intubation:

Despite the initiation of iNO, some patients progressed to requiring intubation. Among patients who progressed to intubation after at least 48 h of iNO treatment, there was no significant difference in respiratory rate, FiO2 requirement, and P/F ratio at 24- and 48 h post-initiation with iNO treatment.

Among patients who were already intubated and iNO was initiated, there was a statistically significant decrease in the respiratory rate at 24 h and 48 h post initiation, as well as in FiO2 requirements and P/F ratio at 24- and 48 h. iNO was also found to decrease PEEP requirements at 24 h and 48 h.

**Fig. 2:**
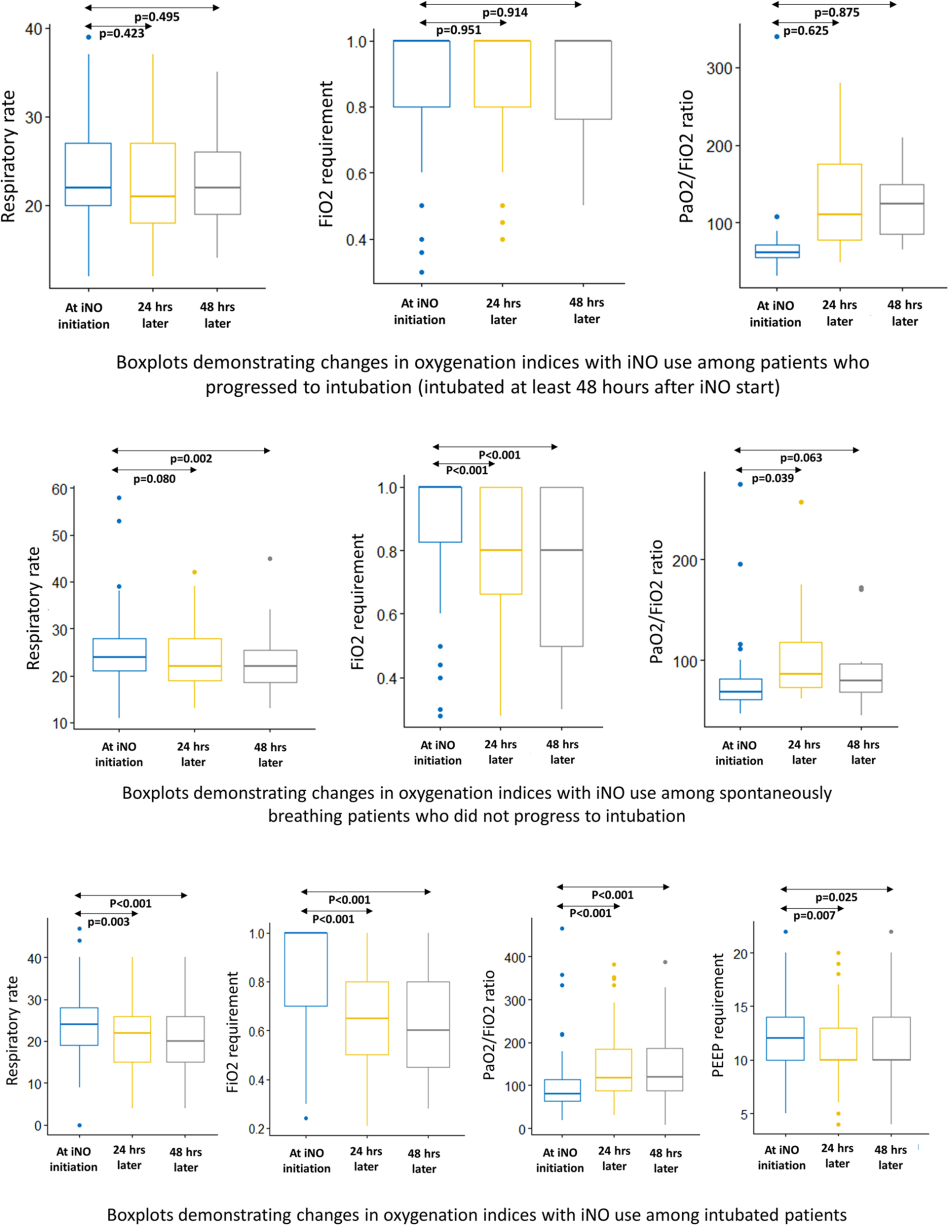
Boxplots demonstrating changes in oxygenation parameters across time with iNO usage among spontaneously breathing and intubated patients

Figure 2 demonstrates changes in oxygenation parameters across time with iNO usage among spontaneously breathing and intubated patients.

### *Patient-centric outcomes (intubation, IMV length, ICU LOS, Hospital LOS, mortality): (*Table 4*)*

**Table 4:** Comparison of hospital outcomes with iNO use in different patient subgroups

	Method	Odds ratio/hazard ratio/estimate	95% CI	*p *value
Spontaneously breathing patients				
Intubation risk	PS-matched	1.08	0.71–1.65	0.717
	IPTW	1.10	0.90–1.33	0.361
In-hospital mortality	PS-matched	0.49	0.32–0.75	**0.001**
	IPTW	0.40	0.26–0.62	** < 0.001**
Creatinine rise	PS-matched	1.75	1.09–2.82	**0.022**
	IPTW	2.18	1.80–2.65	** < 0.001**
CRRT- requirement	PS-matched	1.22	0.58–2.58	0.602
	IPTW	1.05	0.70–1.58	0.801
IMV length	PS-matched	4.73	1.43–8.02	**0.005**
	IPTW	4.84	1.98–7.70	**0.001**
VFD at 28 days	PS-matched	− 3.47	− 6.79; − 0.14	**0.043**
	IPTW	− 3.24	− 5.72; − 0.76	**0.011**
ICU LOS	PS-matched	8.61	5.53–11.69	** < 0.001**
	IPTW	6.49	4.30–8.68	** < 0.001**
ICUFD at 28 days	PS-matched	− 5.18	− 7.27; − 3.09	** < 0.001**
	IPTW	− 4.21	− 5.55; − 2.87	** < 0.001**
Hospital LOS	PS-matched	8.98	5.66–12.30	** < 0.001**
	IPTW	8.76	6.68–10.84	** < 0.001**
Post-intubation cohort				
In-hospital mortality	PS-matched	0.90	0.66–1.24	0.526
	IPTW	0.98	0.73–1.31	0.879
Creatinine rise	PS-matched	3.06	1.33–7.03	**0.008**
	IPTW	3.77	2.56–5.54	** < 0.001**
CRRT- requirement	PS-matched	1.40	0.88–2.22	0.150
	IPTW	1.34	1.03–1.75	**0.029**
IMV length	PS-matched	10.88	7.87–13.90	** < 0.001**
	IPTW	10.69	6.99–14.40	** < 0.001**
Ventilator-free days	PS-matched	− 6.12	− 8.20; − 4.04	** < 0.001**
	IPTW	− 6.70	− 8.28; − 5.12	** < 0.001**
ICU LOS	PS-matched	12.33	8.61–16.04	** < 0.001**
	IPTW	11.57	8.47–14.67	** < 0.001**
ICU free days	PS-matched	− 5.74	− 7.65; − 3.82	** < 0.001**
	IPTW	− 6.20	− 7.66; − 4.74	** < 0.001**
Hospital LOS	PS-matched	12.95	8.33–17.57	** < 0.001**
	IPTW	10.69	6.99–14.40	** < 0.001**

IMV: Invasive mechanical ventilation; NIV: Non-invasive ventilation; HFNC: high-flow nasal cannula; ECMO: extracorporeal membrane oxygenation; CRRT: continuous renal replacement therapy; LOS: length of stay; VFD: ventilator-free days; ICUFD: ICU free days. Statistically significant p-values (<0.05) are highlighted in bold font.

We evaluated relevant patient-centric outcomes both through propensity score (PS) matched and inverse probability treatment weighting (IPTW).

### Spontaneously breathing patients (on HFNC/ NIV)

**Fig. 3:**
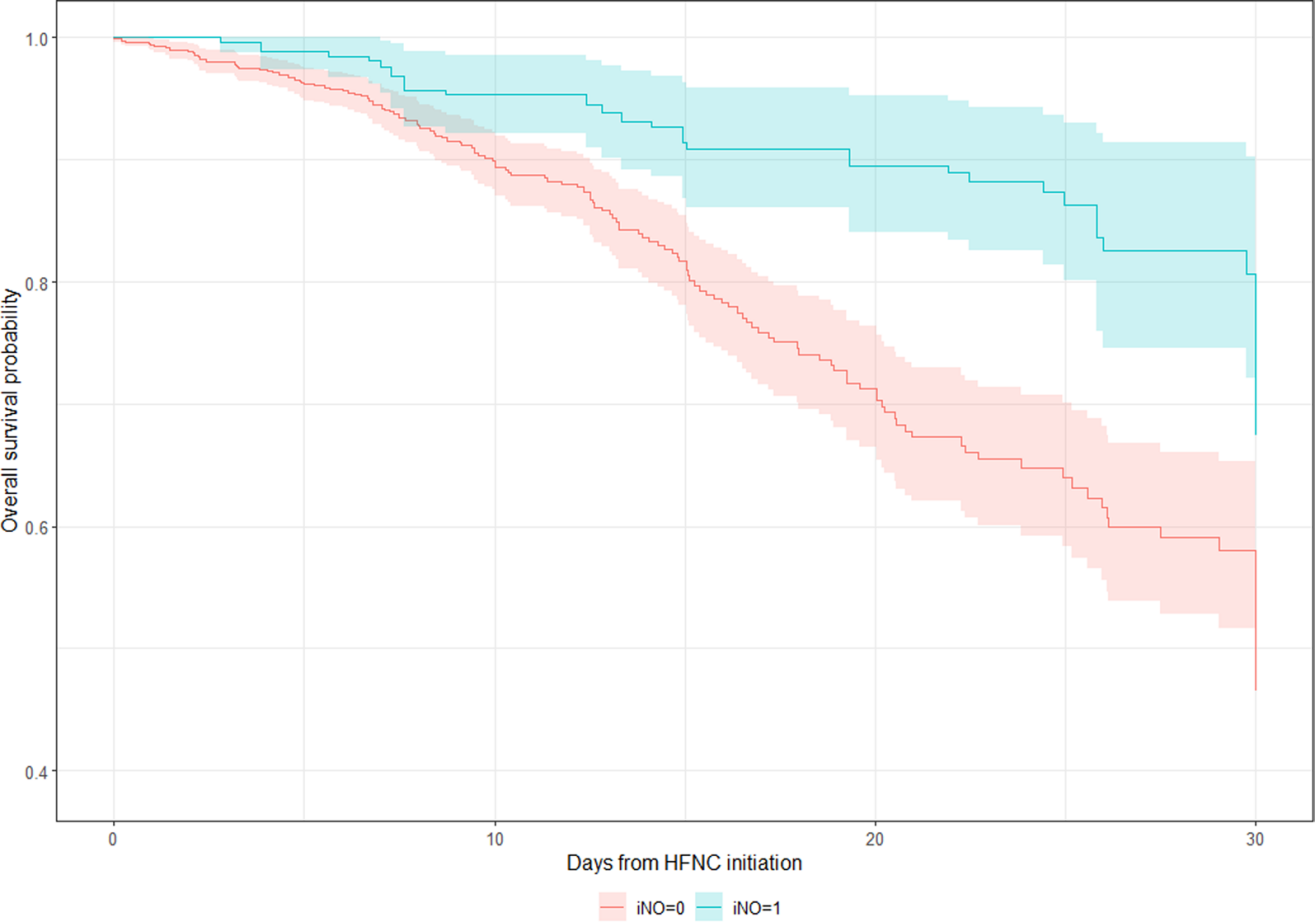
Kaplan Meier curve demonstrating 30-day mortality trends with respect to iNO use in spontaneously breathing patients

On comparing PS-matched groups, spontaneously breathing patients receiving iNO had a longer duration of treatment with HFNC, NIMV, and IMV. They also had a longer ICU and hospital LOS, as well as fewer vent-free or ICU-free days (Table 1). Upon regression analysis, we noted there was no difference in intubation rate, although the iNO-treated patients had a longer duration spent on IMV (PS Estimate: 4.73, CI 1.43–8.02; IPTW Estimate: 4.84, CI 1.98–7.70). Patients receiving iNO also had a longer Hospital length of stay (PS estimate 8.98, CI 5.66—12.30; IPTW estimate 8.76, CI 6.68—10.84) and ICU length of stay (PS Estimate: 8.61, CI 5.53–11.69; IPTW Estimate: 6.49, CI 4.30–8.68). Patients receiving iNO also had fewer vent-free days (PS estimate: − 3.47, CI − 6.79; − 0.14; IPTW estimate: − 3.24, CI − 5.72; − 0.76) and ICU-free days (PS estimate − 5.18, CI − 7.27; − 3.09; IPTW estimate: − 4.21, CI − 5.55; − 2.87). A univariate Cox regression analysis demonstrated decreased mortality risk with iNO use among spontaneously breathing patients upon propensity-matched (HR: 0.49, CI 0.32–0.75) and IPTW analysis (HR 0.40, CI 0.26–0.62) (Table 4). Figure 3 demonstrates the Kaplan–Meier curve for 30-day mortality trends with respect to iNO use in spontaneously breathing patients.

### Mechanically ventilated patients who were initiated on iNO after intubation

**Fig. 4:**
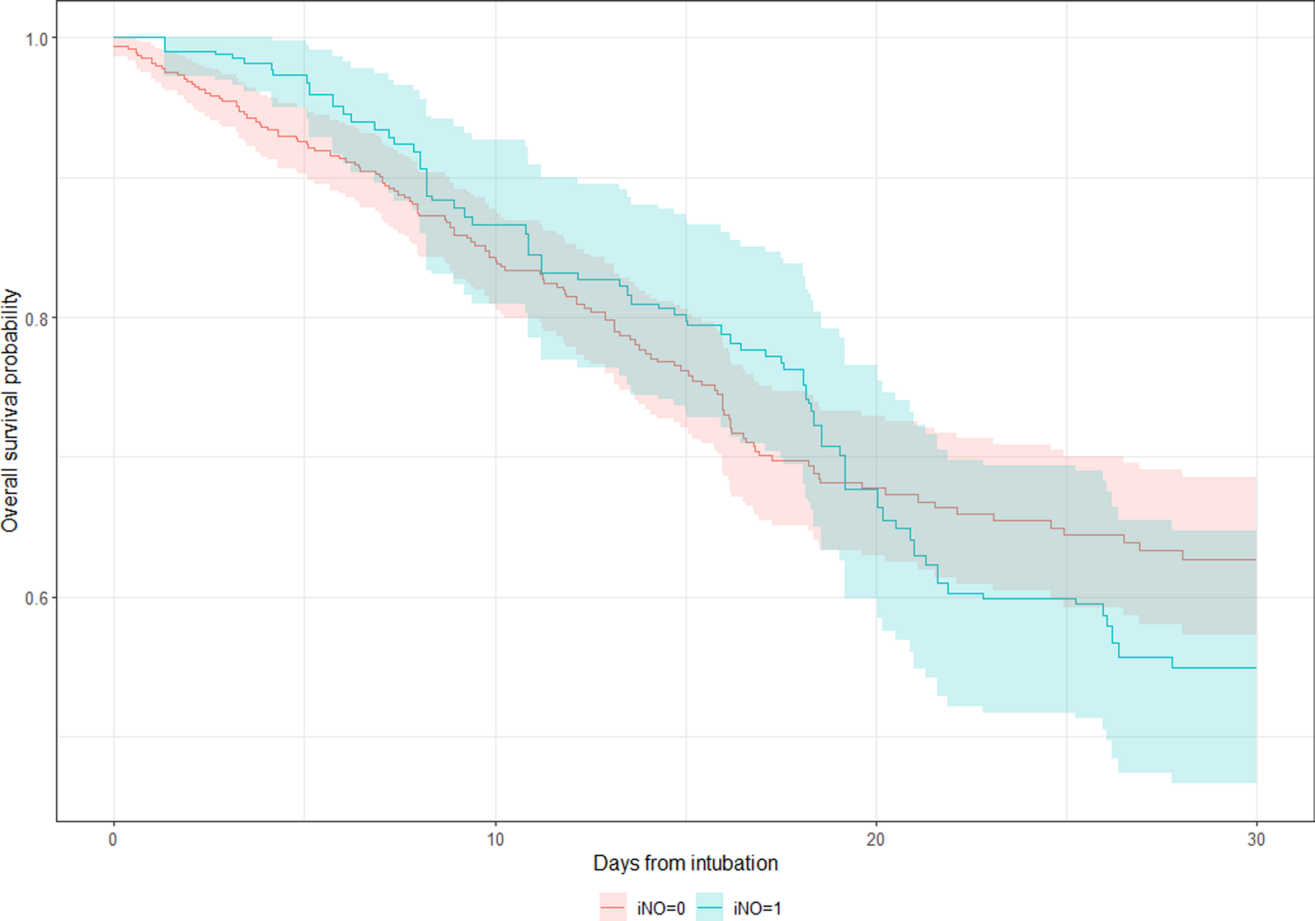
Kaplan Meier curve demonstrating 30-day mortality trends with respect to iNO use in intubated patients

Comparing PS-matched groups, patients receiving iNO after intubation had a longer duration of treatment with HFNC, NIMV, and IMV as well. iNO-treated patients had a longer ICU and hospital LOS. (Table 2) Upon regression analysis, iNO-treated patients had a longer IMV duration (PS-matched estimate: 10.88, CI 7.87–13.90; IPTW estimate: 10.69, CI 6.99–14.40). Intubated patients receiving iNO were more likely to have longer stay in the ICU (PS-matched estimate: 12.33, CI 8.61–16.04; IPTW estimate: 11.57, CI 8.47–14.67) and in the hospital (PS-matched estimate: 12.95, CI 8.33–17.57; IPTW estimate: 10.69, CI 6.99–14.40). Despite a difference in crude in-hospital mortality rate between the groups, there was no difference regarding in-hospital mortality on Cox regression analysis for intubated patients who received iNO (Table 4). Figure 4 demonstrates the Kaplan–Meier curve for 30-day mortality trends with respect to iNO use among intubated patients.

### Events during hospitalization (Creatinine rise, need for CRRT, ECMO use)

#### Spontaneously breathing patients who were supported on HFNC during iNO initiation

We evaluated significant events during the hospitalization, such as creatinine rise, the need for CRRT, and ECMO use. On comparing 2:1 PS-matched groups with similar baseline covariates, spontaneously breathing patients who were treated with iNO demonstrated a higher incidence of creatinine rise (76.2% vs 64.6%, SMD 0.255). However, there was no difference in CRRT requirements. On the other hand, iNO-treated patients had a higher frequency and duration of ECMO use (Table 1).

Our separate PS- weighted and IPTW-based regression analyses demonstrated patients who were started on iNO while spontaneously breathing were more likely to develop a creatinine rise (PS-matched OR 1.75, CI 1.09–2.82; IPTW OR 2.18, CI 1.80–2.65). However, despite higher creatinine rise events, such patients did not show an increased need for CRRT (PS-matched OR 1.22, CI 0.58–2.58; IPTW OR 1.05, CI 0.70–1.58). (Table 4).

### Mechanically ventilated patients who were initiated on iNO after intubation

Similarly, on comparing 2:1 PS-matched intubated patient groups with comparable baseline covariates, iNO use was associated with a higher incidence of creatinine rise (95.2% vs. 86.6%, SMD 0.303) and ECMO use (23.4% vs. 6.9%, SMD 0.474). However, we did not find any difference in the incidence of CRRT use or ECMO duration, although the mean CRRT duration was longer in the iNO group (SMD = 0.330) (Table 2).

In our regression analyses, there was an increased risk of creatinine rise among intubated patients who were initiated on iNO (PS-matched OR 3.06, CI 1.33–7.03; IPTW OR 3.77, CI 2.56–5.54). However, only for IPTW did this translate into a statistically significant difference in CRRT requirement (PS-matched OR 1.40, CI 0.88–2.22; IPTW OR 1.34, CI 1.03–1.75).

### Additional subgroup analysis:

#### Patients progressing to intubation (Supplemental file 1: Table S1)

An additional subgroup analysis evaluated patients who were spontaneously breathing but progressed to intubation at some point during their hospitalization. A 1:1 propensity matched analysis was performed between patients receiving iNO versus those without iNO, to achieve balance in baseline covariates (demographics and comorbidities). Patients undergoing iNO treatment had a longer interval between NIV/HFNC start date and invasive ventilation start date (SMD = 0.389). iNO group also had a longer duration of treatment with HFNC, IMV, and ECMO.

There was no significant difference in the incidence of creatinine rise (SMD = 0.134) between the groups, however, patients in iNO group had a longer duration CRRT (SMD = 0.299). Similarly, iNO-treated patients were more likely to require ECMO, had longer duration of ECMO support, stayed longer in the ICU and in the hospital. There was a trend for lower in-hospital mortality rate among patients receiving iNO treatment, but it did not reach statistical significance (35% vs 43.3%, SMD 0.171).

### Patient undergoing early intubation (Supplemental file 1: Table S2)

An additional analysis was completed of patients treated with HFNC/ NIV who underwent early intubation (intubation within 5 days of non-invasive ventilation). After 1:2 propensity matching for achieving baseline covariate balance, we noted patients receiving iNO had a longer time duration between NIV/HFNC start and progressing to IMV, HFNC duration, NIV duration, days spent on IMV, frequency of ECMO support, ICU and in-hospital length of stay. In the iNO-treated group, the incidence of creatinine rise was higher, but the CRRT duration was lower. Finally, the crude in-hospital mortality was lower among patients who underwent early intubation and were given iNO (28.6% vs 42.9%, SMD 0.302).

### Responders vs non-responders (Supplemental file 1: Table S3)

Our subgroup analysis also evaluated the characteristics of patients who received iNO and responded to those who did not respond to iNO. Patients were defined as responders if they demonstrated improvement in oxygenation status (decrease in FiO2 requirement and/or increase in P/F ratio) within 48 h of iNO initiation. For spontaneously breathing patients, 52 patients were classified as non-responders, while 78 were classified as responders. There was no statistically significant difference (SMD < 0.2) regarding age, race, sex, BMI, or comorbidities. However, the non-responders had a lower COVID-19 vaccination rate (SMD = 0.345) and a higher maximum SOFA score (SMD = 0.465) during hospitalization than the responders. Non-responder patients also showed a higher intubation rate, in-hospital mortality rate, longer IMV duration, NIMV duration, ECMO duration, ICU LOS, and hospital LOS. Furthermore, there was no difference in days of HFNC support or CRRT duration. Patients who responded to iNO had a higher respiratory rate, FiO2 requirement, and PaO2 just prior to iNO initiation. Responders were also started on a relatively higher dose of iNO, but the required maximum iNO dose was relatively lower in them (Supplementary file 1: Table S3).

For intubated patients, there was mostly no significant difference in baseline characteristics and comorbidities between the responders and non-responders, except that responders were more likely to have hypertension, hyperlipidemia, and diabetes. Non-responders had a slightly higher maximum SOFA score (SMD = 0.506). Patients classified as responders required fewer days of IMV, HFNC, and ECMO support. Responders stayed in the ICU (SMD = 0.283) for a shorter period, but there was no difference in overall hospital length of stay (SMD = 0.156). Intubated patients who were responders were started at a higher dose of nitric oxide and titrated to a greater maximum dose. There was no difference in respiratory rate or SpO2 immediately before intubation. Responders did have a higher FiO2 but a lower PaO2 and P/F ratio before iNO initiation (Supplementary file 1: Table S3).

We also explored the difference between intubated and non-intubated groups among those who received iNO while on HFNC. Patients who progressed to intubation despite being treated with iNO while on HFNC support were more likely to be females, have pre-existing comorbidities (diabetes and hypertension), have a higher baseline and maximum SOFA score, but are less likely to be vaccinated against COVID-19. Intubated patients had relatively worse outcomes, such as higher incidence of creatinine rise, use of CRRT, and ECMO usage. They had longer in-hospital and ICU length of stay as well as higher in-hospital mortality rates (Supplementary file 1: Table S4).

## Discussion

Our retrospective multicenter propensity-matched analysis revealed that iNO was associated with improved oxygenation indices at 24 h and 48 h in both non-intubated and mechanically ventilated patients. Interestingly, such differences were not noticed in patients who were initiated on iNO while spontaneously breathing but subsequently progressed to intubation. Among spontaneously breathing patients who were initiated on iNO, our analysis did not show any statistically significant difference in the risk of intubation, although iNO use was associated with significantly increased time interval between non-invasive oxygenation mode initiation and intubation event in such patients. In a time-event analysis, the in-hospital mortality risk was significantly lower with iNO use among spontaneously breathing patients. On the other hand, among patients who were started on iNO while intubated, the risk of mortality was not different compared to the control group. In a subgroup analysis, patients who were ‘responders’ to iNO had a lower intubation rate (among spontaneously breathing patients) and a lower mortality rate (spontaneously breathing and post-intubation).

Alveolar membrane inflammation, ongoing vascular endothelial damage, and the formation of microthrombi in pulmonary vasculature are some of the key mechanisms noticed in COVID-19 and ARDS-related hypoxia. While maintaining adequate oxygenation often requires invasive or non-invasive mechanical ventilation in patients with respiratory failure, additional challenges remain associated with such modalities, such as ventilator-associated complications, sedative requirement, longer hospital stays, increased cost, and healthcare burden. Therefore, from time to time, attempts have been made to decrease oxygen requirement and improve oxygenation indices among such patients to avoid the potential need for invasive ventilatory support [40, 41]. The enhancement of oxygenation through smooth muscle relaxation in the pulmonary vasculature and optimization of ventilation-perfusion matching are known physiological effects of iNO, resulting in an expected improvement in oxygenation indices. In our study, we noted a significantly improved PaO2/FiO2 ratio as well as FiO2 requirement at 24 h and 48 h of iNO initiation in the non-intubated and post-intubation groups.

In our study cohort, the majority (overall 72.93% of patients, 68.24% non-intubated, 82.07% post-intubation, and 44.44% of pre-intubation patients) were found to be ‘responders’ in terms of oxygenation improvement. Such findings align with multiple prior studies focused on the COVID-19 population, including a systematic review and meta-analysis performed by Alqahtani et al., where they reported a cumulative response rate of 66% for patients in terms oxygenation levels following iNO therapy with or without concomitant vasodilators [31]. In studies conducted by Chandel et al. and Tavazzi et al., the percentage of responders was 39% and 25%, respectively [26, 28]. It was interesting, although clinically correlating, to note that most spontaneously breathing patients in our cohort who failed to respond to iNO within 48 h, progressed to intubation during the hospital stay. This may indicate a subset of patients who are less likely to benefit from iNO due to certain patient factors or could represent a group of patients suffering from a higher degree of COVID-19-related systemic inflammatory response that rescue therapy eventually failed to improve oxygenation. We noted that among spontaneous breathers, responders had a higher COVID-19 vaccination rate and a lower intubation rate, ECMO duration, ICU LOS, and mortality rate. This differs from Chandel et al.’s work, which found no difference in intubation among ‘responders,’ although their finding was limited due to a much smaller sample size. On the other hand, keeping with prior study findings, we noted that responders had a higher mean starting dose of iNO yet a lower maximum iNO dose compared to non-responders [26]. This provides some insight into the dose-dependency of iNO responsiveness and should be explored further in future prospective studies.

On the other hand, in the subset of spontaneously breathing patients who progressed to intubation, iNO use was associated with a relatively longer interval between HFNC initiation and intubation, with more than 80% of patients in the iNO group undergoing late intubation. The sustained improvement in oxygenation with rescue iNO therapy could explain such findings. However, the risk of developing patient self-inflicted lung injury (P-SILI) has been reported with increased duration between NIV and IMV, and such risk can be avoided with IMV through strict tidal volume control [42, 43]. From time to time, attempts have been made to identify the optimal timing for intubation among hypoxic COVID-19 patients. A ‘very early intubation’ performed early in the pandemic to avoid viral spread by NIV-induced aerosol generation was refuted by emerging data that showed the safety of non-invasive ventilation and HFNC [44–51]. With the increasing use of non-invasive oxygenation modalities, accumulating evidence suggested that “very late’ or “delayed” were associated with greater mortality than early intubation (within 3–5 days of NIV/ HFNC) [52–57]. Several prediction models have also been developed to identify the risk of NIV failure, and a timely switch to invasive ventilation has been recommended among such patients [58–60]. In order to avoid the confounding effect of delayed intubation on patient outcome, in our subgroup analysis, we looked at all the patients undergoing early intubation. We noted a significantly decreased crude mortality rate with iNO use in this subgroup while using propensity-matched comparison, although sample size was a limiting factor. A more personalized approach, accounting for patients' responsiveness to iNO, should therefore be considered, and a timely switch to invasive ventilation in case of non-improvement could be a more rational approach to improve overall patient outcomes, and future prospective trials can potentially explore such a hypothesis.

Although improvement in oxygenation status has been reported in several past studies, including those focusing on the non-COVID ARDS population, the impact of such improvement on patient-centric outcomes remains unclear. In our propensity-matched and IPTW-analysis, we found no difference in the intubation rate with iNO use among patients supported with HFNC. These findings align with a retrospective study performed by Chandel et al. [26] On the other hand, we observed a noticeable decrease in in-hospital mortality among HFNC-supported patients, which differs from Chandel et al., although their study design was limited by a relatively smaller sample size [26]. Within the intubated cohort, in-hospital mortality did not differ with iNO use, which aligns with two prior systematic reviews and meta-analyses performed on mechanically ventilated non-COVID ARDS patients [12, 61]. While a very limited number of studies have explored the effect of iNO on mortality among intubated COVID-19 patients, the majority were limited by the lack of a comparator group [62, 63]. Al Sulaiman’s study adopted a propensity-matched approach in mechanically ventilated COVID-19 patients and reported no difference in 30-day and in-hospital mortality with iNO use [64]. Moreover, a recent multicentric phase II RCT performed on COVID-19 patients by Di Fenza et al. did not note any difference in the mortality at 28 and 90 days with iNO use, compared to the usual care group, although their study utilized high-dose iNO [30].

These findings induce further research on whether early use of iNO can be beneficial for patient-centric outcomes, particularly in a group of spontaneously breathing patients. In the absence of any apparent benefit in reducing the intubation rate among spontaneously breathing patients, such mortality-lowering effect of iNO is apparently intriguing. While the exact causal mechanism is beyond the scope of this study, we can probably hypothesize that the antiviral and anti-inflammatory properties of iNO could probably have some contribution towards attenuating extrapulmonary ARDS, as reported by prior studies [23, 30].

The development of AKI in the background of COVID-19 has been well-studied and linked to virus-induced systemic inflammation. However, renal dysfunction is also one of the potential adverse effects that have been linked with iNO use in the ARDS population. A 2016 Cochrane Review of the Clinical Trials reported a pooled relative risk of 1.59 (95% CI 1.17–2.16; I2 = 0%) for the development of renal impairment in patients receiving iNO, and the evidence was upgraded from moderate to high quality [12]. In a separate meta-analysis performed by Wang et al., iNO was again found to be a risk factor for AKI in ARDS patients, but it reduced the incidence of AKI in patients undergoing cardiac surgery, thereby proposing a disease-specific effect on renal function with iNO use [65]. In our study, we noted a consistent increase in the incidence of creatinine rise associated with iNO use in spontaneously breathing and intubated COVID-19 patients. These findings align with Al Sulaiman et al.’s study but not with Chandel et al.’s work—both focused on the COVID-19 population [26, 64]. Moreover, we did not find any statistically significant difference in the requirement for CRRT or duration of CRRT with iNO use, aligning with prior literature. Therefore, careful consideration should probably be made about the potential for transient renal dysfunction upon iNO use in the COVID-19 population.

In summary, this large retrospective multicentre study demonstrated a potential beneficial effect of iNO, particularly reducing mortality in spontaneously breathing COVID-19 patients. While it may delay the time to intubation, possibly through improvement in oxygenation indices, we did not notice a decrease in intubation rate with iNO use. The beneficial role of iNO towards patient-centric outcomes was not apparent in the intubated cohort, but iNO did show a consistent increase in oxygenation indices, thereby highlighting its potential rescue role in refractory hypoxemia. Moreover, there was a consistently high incidence of creatinine rise among patients receiving iNO. Therefore, further large-scale prospective studies are needed to fully understand its utility.

### Strengths and limitations

This study has several strengths. First, we included intubated as well as spontaneously breathing patients and evaluated the impact of nitric oxide separately in both patient groups. Each group comprised a relatively larger sample size than the existing literature, which held true even after propensity matching. Second, including two different patient subcategories (spontaneously breathing and post-intubation) from the same multicentric clinical establishments helps avoid heterogeneity in the standard of clinical care, treatment approaches, and patient characteristics. Prior studies have looked at both patient subgroups but in different patient populations, raising questions about the comparability of findings. Third, adopting multiple complementary statistical strategies has also allowed us to minimize the risk of bias across two groups and evaluate the consistency of findings across varying sample sizes. Finally, the current study is one of the few studies that have explored the patient-outcome-centric effects of iNO in the ARDS population, keeping in line with current best practices after the introduction of the ADSNet protocol, where there is a paucity of data.

On the other hand, this study has certain limitations that are worth noting. The retrospective design, even though supported by rigorous methodology, denies us from establishing causality. COVID-19 is a multisystemic inflammatory disease state and often presents with phenotypically different inflammatory responses despite similar patient characteristics. Accounting for such confounding factors is often beyond the scope of traditional statistical approaches. Certain complications like creatinine rise are known adverse outcomes associated with COVID-19 disease itself, and the associations between such complications with iNO could at least be partially affected by confounders. Second, we considered any creatinine rise developed during the hospitalization period that may not have a temporal association with iNO exposure in the iNO cohort. On the other hand, the difference in creatinine rise could also be confounded by reduced or delayed mortality in the iNO group as they spent a longer time in the hospital. However, in order to avoid the risk of introducing bias, we opted not to adjust for post-baseline covariates. Due to insufficient data on hourly urine output in our database, we were not able to utilize full KDIGO criteria to make formal AKI diagnoses. We also chose to use the lowest creatinine during hospitalization as baseline creatinine, which is often considered a crude approach and denies us to capture patients presenting with AKI at admission. Third, although we collected the starting and maximum dose of iNO, we did not perform a dose or duration-dependent outcome analysis in the iNO group due to the dynamic nature of dosing and its fluctuation based on the patient’s clinical response. The study participant received a low-dose iNO treatment, whereas prior studies have studied variable responses with different dosing regimens [66, 67]. In that aspect, our findings may have limited generalizability for different dosing practices. Fourth, the use of iNO was semi-protocolized per institutional practice guidelines but was often affected by clinician judgment based on the likelihood of treatment success or unmeasured patient factors (such as goals of care, comorbidity burden, etc.) [35]. Despite adopting a robust methodology, the effects of these confounding factors sometimes cannot be overcome. Finally, many patients in our cohort were supported by adjunctive measures for decreasing inflammation (e.g., corticosteroids, interleukin inhibitors, antivirals, etc.) or improving the oxygenation (e.g., prone positioning, prostaglandin analogs, etc.), which could be potential confounders. Although we did not specifically include those, we believe such interventions were equally offered to both patient categories whenever appropriate based on clinician judgment.

## Conclusions

In this multicentric retrospective study on hospitalized COVID-19 patients, the use of iNO was associated with significant improvements in oxygenation indices among both spontaneously and intubated patients. While iNO use was not associated with intubation rate reduction, it was associated with lowered in-hospital mortality among spontaneously breathing patients but not in the post-intubation cohort. In both patient cohorts, iNO was associated with an increased risk of creatinine rise.

## Supplementary Information

Supplemental material 1

## Data Availability

Due to institutional policies, data is available upon reasonable request addressed to the corresponding author.

## Abbreviations

COVID-19: Coronavirus Disease 2019
AKI: Acute kidney injury
ARDS: Acute respiratory distress syndrome
iNO: Inhaled nitric oxide
PS: Propensity score
IPTW: Inverse propensity of treatment weighted
BMI: Body mass index
ICU: Intensive care unit
SCCM: Society of critical care medicine
VIRUS: Viral infection and respiratory illness universal study
CAD: Coronary artery disease
CHF: Congestive heart failure
COPD: Chronic obstructive pulmonary disease
HTN: Hypertension
CKD: Chronic kidney disease
BMI: Body mass index
SBP: Systolic blood pressure
DBP: Diastolic blood pressure
SpO2: Peripheral capillary oxygen saturation
SOFA: Sequential organ failure assessment
BUN: Blood urea nitrogen
SGOT: Serum glutamic-oxaloacetic transaminase
SGPT: Serum glutamic-pyruvic transaminase
HFNC: High flow nasal cannula
NIV: Non-invasive ventilation
IMV: Intermittent mandatory ventilation
ECMO: Extracorporeal membrane oxygenation
LOS: Length of stay

## References

[1] Bernard GR, Artigas A, Brigham KL, Carlet J, Falke K, Hudson L, et al. The American-European consensus conference on ARDS. Definitions, mechanisms, relevant outcomes, and clinical trial coordination. Am J Respir Crit Care Med. 1994;149(3 Pt 1):818–24. 7509706 10.1164/ajrccm.149.3.7509706

[2] Kamo T, Tasaka S, Suzuki T, Asakura T, Suzuki S, Yagi K, et al. Prognostic values of the Berlin definition criteria, blood lactate level, and fibroproliferative changes on high-resolution computed tomography in ARDS patients. BMC Pulm Med. 2019;19(1):37. 30744598 10.1186/s12890-019-0803-0 PMC6371514

[3] ARDS Definition Task Force, Ranieri VM, Rubenfeld GD, Thompson BT, Ferguson ND, Caldwell E, et al. Acute respiratory distress syndrome: the Berlin Definition. JAMA. 2012;307(23):2526–33. 10.1001/jama.2012.5669 22797452

[4] Rossaint R, Falke KJ, López F, Slama K, Pison U, Zapol WM. Inhaled nitric oxide for the adult respiratory distress syndrome. N Engl J Med. 1993;328(6):399–405. 8357359 10.1056/NEJM199302113280605

[5] Creagh-Brown BC, Griffiths MJD, Evans TW. Bench-to-bedside review: Inhaled nitric oxide therapy in adults. Crit Care Lond Engl. 2009;13(3):221. 10.1186/cc7734 PMC2717403 19519946

[6] Gries A, Bode C, Peter K, Herr A, Böhrer H, Motsch J, et al. Inhaled nitric oxide inhibits human platelet aggregation, P-selectin expression, and fibrinogen binding in vitro and in vivo. Circulation. 1998;97(15):1481–7. 9576429 10.1161/01.cir.97.15.1481

[7] Gries A, Herr A, Motsch J, Holzmann A, Weimann J, Taut F, et al. Randomized, placebo-controlled, blinded and cross-matched study on the antiplatelet effect of inhaled nitric oxide in healthy volunteers. Thromb Haemost. 2000;83(2):309–15. 10739391

[8] Hunt JL, Bronicki RA, Anas N. Role of inhaled nitric oxide in the management of severe acute respiratory distress syndrome. Front Pediatr. 2016;4:74. 27532031 10.3389/fped.2016.00074 PMC4970488

[9] Karam O, Gebistorf F, Wetterslev J, Afshari A. The effect of inhaled nitric oxide in acute respiratory distress syndrome in children and adults: a cochrane systematic review with trial sequential analysis. Anaesthesia. 2017;72(1):106–17. 27762438 10.1111/anae.13628

[10] Adhikari NKJ, Burns KEA, Friedrich JO, Granton JT, Cook DJ, Meade MO. Effect of nitric oxide on oxygenation and mortality in acute lung injury: systematic review and meta-analysis. BMJ. 2007;334(7597):779. 17383982 10.1136/bmj.39139.716794.55 PMC1852043

[11] MekontsoDessap A, Papazian L, Schaller M, Nseir S, Megarbane B, Haudebourg L, et al. Inhaled nitric oxide in patients with acute respiratory distress syndrome caused by COVID-19: treatment modalities, clinical response, and outcomes. Ann Intensive Care. 2023;13(1):57. 37368036 10.1186/s13613-023-01150-9 PMC10299982

[12] Gebistorf F, Karam O, Wetterslev J, Afshari A. Inhaled nitric oxide for acute respiratory distress syndrome (ARDS) in children and adults. Cochrane Database Syst Rev. 2016;2016(6):CD002787. 10.1002/14651858.CD002787.pub3 PMC6464789 27347773

[13] Wu C, Chen X, Cai Y, Xia J, Zhou X, Xu S, et al. Risk factors associated with acute respiratory distress syndrome and death in patients with coronavirus disease 2019 Pneumonia in Wuhan, China. JAMA Intern Med. 2020;180(7):934. 32167524 10.1001/jamainternmed.2020.0994 PMC7070509

[14] Lim ZJ, Subramaniam A, Ponnapa Reddy M, Blecher G, Kadam U, Afroz A, et al. Case fatality rates for patients with COVID-19 requiring invasive mechanical ventilation. A Meta-analysis. Am J Respir Crit Care Med. 2021;203(1):54–66. 33119402 10.1164/rccm.202006-2405OC PMC7781141

[15] Oliveira E, Parikh A, Lopez-Ruiz A, Carrilo M, Goldberg J, Cearras M, et al. ICU outcomes and survival in patients with severe COVID-19 in the largest health care system in central Florida. PLoS ONE. 2021;16(3): e0249038. 33765049 10.1371/journal.pone.0249038 PMC7993561

[16] Wang Y, Lu X, Li Y, Chen H, Chen T, Su N, et al. Clinical course and outcomes of 344 intensive care patients with COVID-19. Am J Respir Crit Care Med. 2020;201(11):1430–4. 32267160 10.1164/rccm.202003-0736LE PMC7258632

[17] Yang X, Yu Y, Xu J, Shu H, Xia J, Liu H, et al. Clinical course and outcomes of critically ill patients with SARS-CoV-2 pneumonia in Wuhan, China: a single-centered, retrospective, observational study. Lancet Respir Med. 2020;8(5):475–81. 32105632 10.1016/S2213-2600(20)30079-5 PMC7102538

[18] Aslan A, Aslan C, Zolbanin NM, Jafari R. Acute respiratory distress syndrome in COVID-19: possible mechanisms and therapeutic management. Pneumonia Nathan Qld. 2021;13(1):14. 34872623 10.1186/s41479-021-00092-9 PMC8647516

[19] Alhazzani W, Møller MH, Arabi YM, Loeb M, Gong MN, Fan E, et al. Surviving sepsis campaign: guidelines on the management of critically ill adults with coronavirus disease 2019 (COVID-19). Intensive Care Med. 2020;46(5):854–87. 32222812 10.1007/s00134-020-06022-5 PMC7101866

[20] Matthay MA, Aldrich JM, Gotts JE. Treatment for severe acute respiratory distress syndrome from COVID-19. Lancet Respir Med. 2020;8(5):433–4. 32203709 10.1016/S2213-2600(20)30127-2 PMC7118607

[21] Feng WX, Yang Y, Wen J, Liu YX, Liu L, Feng C. Implication of inhaled nitric oxide for the treatment of critically ill COVID-19 patients with pulmonary hypertension. ESC Heart Fail. 2021;8(1):714–8. 33205620 10.1002/ehf2.13023 PMC7753827

[22] Kobayashi J, Murata I. Nitric oxide inhalation as an interventional rescue therapy for COVID-19-induced acute respiratory distress syndrome. Ann Intensive Care. 2020;10(1):61. 32436029 10.1186/s13613-020-00681-9 PMC7238394

[23] Åkerström S, Mousavi-Jazi M, Klingström J, Leijon M, Lundkvist Å, Mirazimi A. Nitric oxide inhibits the replication cycle of severe acute respiratory syndrome coronavirus. J Virol. 2005;79(3):1966–9. 15650225 10.1128/JVI.79.3.1966-1969.2005 PMC544093

[24] Taylor RW, Zimmerman JL, Dellinger RP, Straube RC, Criner GJ, Davis K, et al. Low-dose inhaled nitric oxide in patients with acute lung injury: a randomized controlled trial. JAMA. 2004;291(13):1603–9. 15069048 10.1001/jama.291.13.1603

[25] Abman SH, Fox NR, Malik MI, Kelkar SS, Corman SL, Rege S, et al. Real-world use of inhaled nitric oxide therapy in patients with COVID-19 and mild-to-moderate acute respiratory distress syndrome. Drugs Context. 2022;11:2022–1–4. 10.7573/dic.2022-1-4 PMC9007062 35462641

[26] Chandel A, Patolia S, Ahmad K, Aryal S, Brown AW, Sahjwani D, et al. Inhaled nitric oxide via high-flow nasal cannula in patients with acute respiratory failure related to COVID-19. Clin Med Insights Circ Respir Pulm Med. 2021;15:11795484211047064. 34602831 10.1177/11795484211047065 PMC8485265

[27] SafaeeFakhr B, Di Fenza R, Gianni S, Wiegand SB, Miyazaki Y, Araujo Morais CC, et al. Inhaled high dose nitric oxide is a safe and effective respiratory treatment in spontaneous breathing hospitalized patients with COVID-19 pneumonia. Nitric Oxide Biol Chem. 2021;1(116):7–13. 10.1016/j.niox.2021.08.003 PMC8361002 34400339

[28] Tavazzi G, Pozzi M, Mongodi S, Dammassa V, Romito G, Mojoli F. Inhaled nitric oxide in patients admitted to intensive care unit with COVID-19 pneumonia. Crit Care Lond Engl. 2020;24(1):508. 10.1186/s13054-020-03222-9 PMC7429937 32807220

[29] Ziehr DR, Alladina J, Wolf ME, Brait KL, Malhotra A, La Vita C, et al. Respiratory physiology of prone positioning with and without inhaled nitric oxide across the coronavirus disease 2019 acute respiratory distress syndrome severity spectrum. Crit Care Explor. 2021;3(6): e0471. 34151287 10.1097/CCE.0000000000000471 PMC8208401

[30] Di Fenza R, Shetty NS, Gianni S, Parcha V, Giammatteo V, SafaeeFakhr B, et al. High-dose inhaled nitric oxide in acute hypoxemic respiratory failure due to COVID-19: a multicenter phase II Trial. Am J Respir Crit Care Med. 2023;208(12):1293–304. 37774011 10.1164/rccm.202304-0637OC PMC10765403

[31] Alqahtani JS, Aldhahir AM, Al Ghamdi SS, AlBahrani S, AlDraiwiesh IA, Alqarni AA, et al. Inhaled nitric oxide for clinical management of COVID-19: a systematic review and meta-analysis. Int J Environ Res Public Health. 2022;19(19):12803. 36232100 10.3390/ijerph191912803 PMC9566710

[32] Walkey AJ, Sheldrick RC, Kashyap R, Kumar VK, Boman K, Bolesta S, et al. Guiding principles for the conduct of observational critical care research for coronavirus disease 2019 pandemics and beyond: the society of critical care medicine discovery viral infection and respiratory illness universal study registry. Crit Care Med. 2020;48(11):e1038–44. 32932348 10.1097/CCM.0000000000004572 PMC7540620

[33] Walkey AJ, Sheldrick RC, Kashyap R, Kumar VK, Boman K, Bolesta S, et al. Guiding principles for the conduct of observational critical care research for coronavirus disease 2019 pandemics and beyond: the society of critical care medicine discovery viral infection and respiratory illness universal study registry. Crit Care Med. 2020;48(11):e1038–44. 32932348 10.1097/CCM.0000000000004572 PMC7540620

[34] Domecq JP, Lal A, Sheldrick CR, Kumar VK, Boman K, Bolesta S, et al. Outcomes of patients with coronavirus disease 2019 receiving organ support therapies: the international viral infection and respiratory illness universal study registry. Crit Care Med. 2021;49(3):437–48. 33555777 10.1097/CCM.0000000000004879 PMC9520995

[35] Yarrarapu SNS, Bansal P, Abia-Trujillo D, Cusick A, Melody M, Moktan V, et al. V.I.T.A.M. in COVID 19: A Systematic Approach to a Global Pandemic. Clin Med Insights Circ Respir Pulm Med. 2021;15:11795484211047432. 10.1177/11795484211047432 PMC8493324 34629922

[36] Section 2: AKI definition. Kidney Int Suppl. 2012;2(1):19–36. 10.1038/kisup.2011.32 PMC4089595 25018918

[37] Ho DE, Imai K, King G, Stuart EA. **MatchIt** : Nonparametric Preprocessing for Parametric Causal Inference. J Stat Softw [Internet]. 2011 [cited 2024 May 4];42(8). http://www.jstatsoft.org/v42/i08/

[38] Seppala LJ, van de Loo B, Schut M, van Schoor NM, Stricker BH, Kenny RA, et al. A propensity score matched approach to assess the associations of commonly prescribed medications with fall risk in a large harmonized cohort of older ambulatory persons. Drugs Aging. 2021;38(9):797–805. 34224104 10.1007/s40266-021-00876-0 PMC8419131

[39] Austin PC. The use of propensity score methods with survival or time-to-event outcomes: reporting measures of effect similar to those used in randomized experiments. Stat Med. 2014;33(7):1242–58. 24122911 10.1002/sim.5984 PMC4285179

[40] Longobardo A, Montanari C, Shulman R, Benhalim S, Singer M, Arulkumaran N. Inhaled nitric oxide minimally improves oxygenation in COVID-19 related acute respiratory distress syndrome. Br J Anaesth. 2021;126(1):e44–6. 33138964 10.1016/j.bja.2020.10.011 PMC7556790

[41] Grieco DL, Maggiore SM, Roca O, Spinelli E, Patel BK, Thille AW, et al. Non-invasive ventilatory support and high-flow nasal oxygen as first-line treatment of acute hypoxemic respiratory failure and ARDS. Intensive Care Med. 2021;47(8):851–66. 34232336 10.1007/s00134-021-06459-2 PMC8261815

[42] Brochard L, Slutsky A, Pesenti A. Mechanical ventilation to minimize progression of lung injury in acute respiratory failure. Am J Respir Crit Care Med. 2017;195(4):438–42. 27626833 10.1164/rccm.201605-1081CP

[43] Grotberg JC, Kraft BD. Timing of intubation in COVID-19: when it is too early and when it is too late. Crit Care Explor. 2023;5(2): e0863. 36817964 10.1097/CCE.0000000000000863 PMC9928829

[44] Villarreal-Fernandez E, Patel R, Golamari R, Khalid M, DeWaters A, Haouzi P. A plea for avoiding systematic intubation in severely hypoxemic patients with COVID-19-associated respiratory failure. Crit Care Lond Engl. 2020;24(1):337. 10.1186/s13054-020-03063-6 PMC7291186 32532318

[45] World Health Organization. Clinical management of severe acute respiratory infection (SARI) when COVID-19 disease is suspected: interim guidance, 13 March 2020. 2020; https://apps.who.int/iris/handle/10665/331446

[46] Cheung JCH, Ho LT, Cheng JV, Cham EYK, Lam KN. Staff safety during emergency airway management for COVID-19 in Hong Kong. Lancet Respir Med. 2020;8(4): e19. 32105633 10.1016/S2213-2600(20)30084-9 PMC7128208

[47] Marini JJ, Gattinoni L. Management of COVID-19 respiratory distress. JAMA. 2020;323(22):2329–30. 32329799 10.1001/jama.2020.6825

[48] Brown CA, Mosier JM, Carlson JN, Gibbs MA. Pragmatic recommendations for intubating critically ill patients with suspected COVID-19. J Am Coll Emerg Physicians Open. 2020;1(2):80–4. 32427182 10.1002/emp2.12063 PMC7228350

[49] Docherty AB, Mulholland RH, Lone NI, Cheyne CP, De Angelis D, Diaz-Ordaz K, et al. Changes in in-hospital mortality in the first wave of COVID-19: a multicentre prospective observational cohort study using the WHO Clinical Characterisation Protocol UK. Lancet Respir Med. 2021;9(7):773–85. 34000238 10.1016/S2213-2600(21)00175-2 PMC8121531

[50] Parish AJ, West JR, Caputo ND, Janus TM, Yuan D, Zhang J, et al. Early intubation and increased coronavirus disease 2019 mortality: A propensity score-matched retrospective cohort study. Crit Care Explor. 2021;3(6): e0452. 34151281 10.1097/CCE.0000000000000452 PMC8208412

[51] Perkins GD, Ji C, Connolly BA, Couper K, Lall R, Baillie JK, et al. Effect of noninvasive respiratory strategies on intubation or mortality among patients with acute hypoxemic respiratory failure and COVID-19: The RECOVERY-RS randomized clinical trial. JAMA. 2022;327(6):546–58. 35072713 10.1001/jama.2022.0028 PMC8787685

[52] Camous L, Pommier JD, Martino F, Tressieres B, Demoule A, Valette M. Very late intubation in COVID-19 patients: a forgotten prognosis factor? Crit Care Lond Engl. 2022;26(1):89. 10.1186/s13054-022-03966-6 PMC8976275 35366941

[53] Karagiannidis C, Hentschker C, Westhoff M, Weber-Carstens S, Janssens U, Kluge S, et al. Observational study of changes in utilization and outcomes in mechanical ventilation in COVID-19. PLoS ONE. 2022;17(1): e0262315. 35030205 10.1371/journal.pone.0262315 PMC8759661

[54] González J, Benítez ID, de Gonzalo-Calvo D, Torres G, de Batlle J, Gómez S, et al. Impact of time to intubation on mortality and pulmonary sequelae in critically ill patients with COVID-19: a prospective cohort study. Crit Care Lond Engl. 2022;26(1):18. 10.1186/s13054-021-03882-1 PMC8744383 35012662

[55] Polok K, Fronczek J, Artigas A, Flaatten H, Guidet B, De Lange DW, et al. Noninvasive ventilation in COVID-19 patients aged ≥ 70 years-a prospective multicentre cohort study. Crit Care Lond Engl. 2022;26(1):224. 10.1186/s13054-022-04082-1 PMC9305028 35869557

[56] Hyman JB, Leibner ES, Tandon P, Egorova NN, Bassily-Marcus A, Kohli-Seth R, et al. Timing of intubation and in-hospital mortality in patients with coronavirus disease 2019. Crit Care Explor. 2020;2(10): e0254. 33134945 10.1097/CCE.0000000000000254 PMC7587418

[57] Vera M, Kattan E, Born P, Rivas E, Amthauer M, Nesvadba A, et al. Intubation timing as determinant of outcome in patients with acute respiratory distress syndrome by SARS-CoV-2 infection. J Crit Care. 2021;65:164–9. 34166852 10.1016/j.jcrc.2021.06.008 PMC8216652

[58] Shu W, Guo S, Yang F, Liu B, Zhang Z, Liu X, et al. Association between ARDS etiology and risk of noninvasive ventilation failure. Ann Am Thorac Soc. 2022;19(2):255–63. 34288830 10.1513/AnnalsATS.202102-161OC

[59] Roca O, Caralt B, Messika J, Samper M, Sztrymf B, Hernández G, et al. An index combining respiratory rate and oxygenation to predict outcome of nasal high-flow therapy. Am J Respir Crit Care Med. 2019;199(11):1368–76. 30576221 10.1164/rccm.201803-0589OC

[60] Roca O, Messika J, Caralt B, García-de-Acilu M, Sztrymf B, Ricard JD, et al. Predicting success of high-flow nasal cannula in pneumonia patients with hypoxemic respiratory failure: The utility of the ROX index. J Crit Care. 2016;35:200–5. 27481760 10.1016/j.jcrc.2016.05.022

[61] Adhikari NKJ, Dellinger RP, Lundin S, Payen D, Vallet B, Gerlach H, et al. Inhaled nitric oxide does not reduce mortality in patients with acute respiratory distress syndrome regardless of severity: systematic review and meta-analysis*. Crit Care Med. 2014;42(2):404–12. 24132038 10.1097/CCM.0b013e3182a27909

[62] Parikh R, Wilson C, Weinberg J, Gavin D, Murphy J, Reardon CC. Inhaled nitric oxide treatment in spontaneously breathing COVID-19 patients. Ther Adv Respir Dis. 2020;14:175346662093351. 10.1177/1753466620933510 PMC7298422 32539647

[63] Ferrari M, Santini A, Protti A, Andreis DT, Iapichino G, Castellani G, et al. Inhaled nitric oxide in mechanically ventilated patients with COVID-19. J Crit Care. 2020;60:159–60. 32814271 10.1016/j.jcrc.2020.08.007 PMC7417286

[64] Al Sulaiman K, Korayem GB, Altebainawi AF, Al Harbi S, Alissa A, Alharthi A, et al. Evaluation of inhaled nitric oxide (iNO) treatment for moderate-to-severe ARDS in critically ill patients with COVID-19: a multicenter cohort study. Crit Care. 2022;26(1):304. 36192801 10.1186/s13054-022-04158-y PMC9527729

[65] Wang J, Cong X, Miao M, Yang Y, Zhang J. Inhaled nitric oxide and acute kidney injury risk: a meta-analysis of randomized controlled trials. Ren Fail. 2021;43(1):281–90. 33494652 10.1080/0886022X.2021.1873805 PMC7850389

[66] Zapol WM, Hurford WE. Inhaled Nitric Oxide in Adult Respiratory Distress Syndrome and Other Lung Diseases. In: Advances in Pharmacology [Internet]. Elsevier; 1994 [cited 2024 Jul 6]. p. 513–30. https://linkinghub.elsevier.com/retrieve/pii/S1054358908606390 10.1016/s1054-3589(08)60639-0 7873435

[67] Gerlach H, Keh D, Semmerow A, Busch T, Lewandowski K, Pappert DM, et al. Dose-response characteristics during long-term inhalation of nitric oxide in patients with severe acute respiratory distress syndrome: a prospective, randomized, controlled study. Am J Respir Crit Care Med. 2003;167(7):1008–15. 12663340 10.1164/rccm.2108121

