# Supplementary material for: Impact of low dose inhaled nitric oxide treatment in spontaneously breathing and intubated COVID-19 patients: a retrospective propensity-matched study
Source: Crit Care. 2024 Oct 25;28:344. doi: 10.1186/s13054-024-05093-w (PMC11515277; doi:10.1186/s13054-024-05093-w)
Supplement: Supplementary file 1 — Supplemental material 1 [file 13054_2024_5093_MOESM1_ESM.docx]

Supplementary Figure 1: Demonstration of covariate balance in HFNC/ intubated patients before and after propensity weight matching


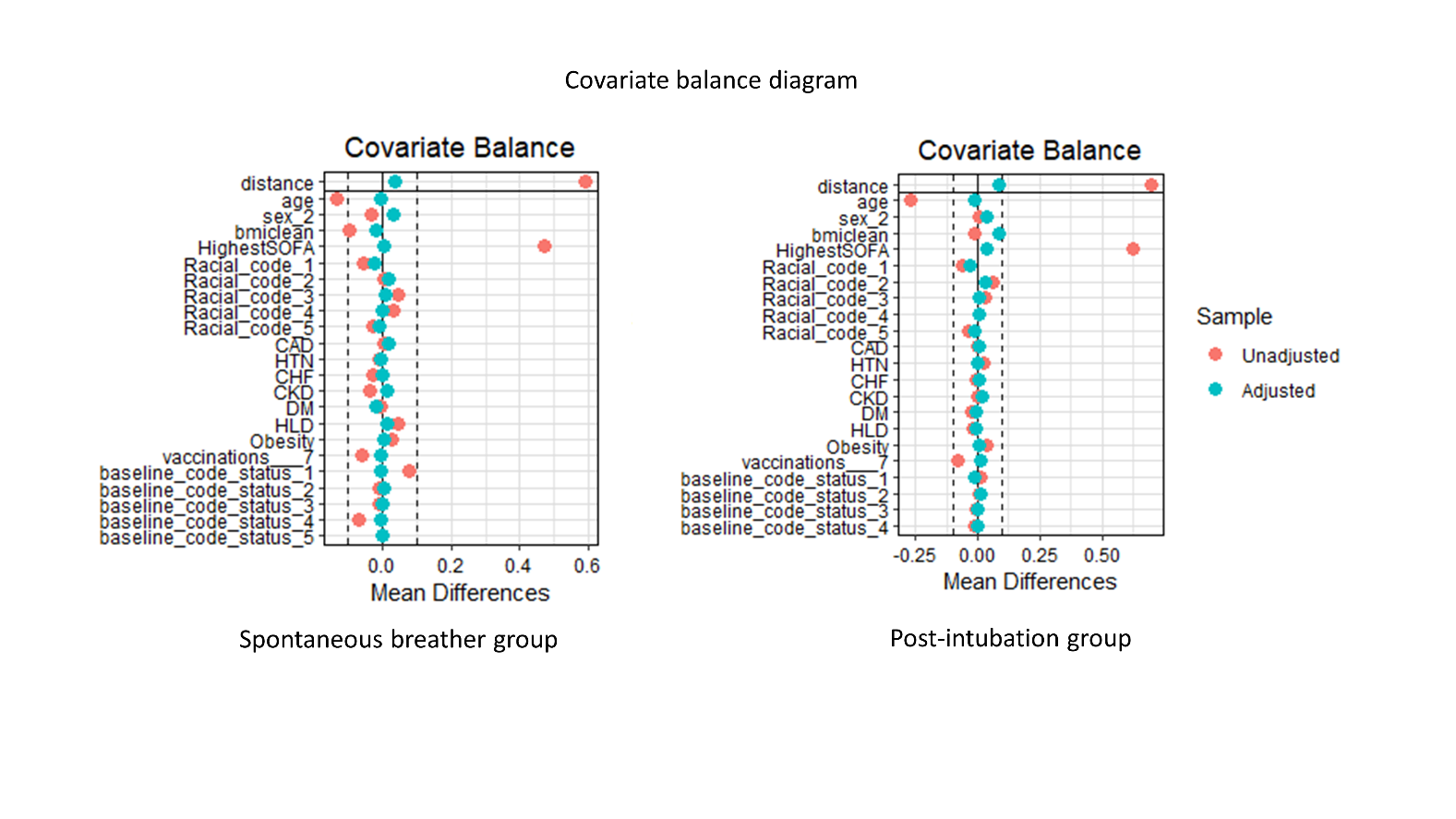


Supplementary Table 1: comparison among HFNC +/- iNO treated patients who progressed to intubation

|  | Before Matching | | | After Matching | | | |
| --- | --- | --- | --- | --- | --- | --- | --- |
| Variables | No iNO used (mean (SD)) (n=301) | iNO used (n=68) | SMD | No iNO used (n=60) | iNO used (n=60) | SMD |  |
| Age | 62.25 (14.60) | 63.43 (11.94) | 0.088 | 64.48 (11.15) | 63.60 (12.36) | 0.075 |  |
| Female | 102 (33.9) | 28 (41.2) | 0.151 | 26 (43.3) | 23 (38.3) | 0.102 |  |
| Race | | | **0.477** |  | | 0.185 |  |
| Caucasian | 256 (85.0) | 49 (72.1) |  | 45 (75.0) | 43 (71.7) |  |  |
| American Indian/Alaska Native/ Pacific Islander | 4 (1.3) | 4 (5.9) |  | 2 (3.3) | 3 (5.0) |  |  |
| African American | 3 (1.0) | 5 (7.4) |  | 2 (3.3) | 4 (6.7) |  |  |
| Asian | 13 (4.3) | 6 (8.8) |  | 6 (10.0) | 6 (10.0) |  |  |
| Mixed/Other/Unknown | 25 (8.3) | 4 (5.9) |  | 5 (8.3) | 4 (6.7) |  |  |
| BMI | 33.68 (8.50) | 30.93 (5.85) | **0.377** | 31.54 (5.13) | 30.87 (5.74) | 0.123 |  |
| NIV to IMV days | 3.42 (3.94) | 8.78 (9.33) | **0.749** | 6.02 (4.55) | 8.98 (9.78) | **0.389** |  |
| Highest SOFA | 12.41 (2.67) | 12.62 (2.68) | 0.081 | 12.75 (2.52) | 12.58 (2.75) | 0.063 |  |
| SOFA | 6.03 (2.80) | 5.98 (2.17) | 0.017 | 5.60 (2.16) | 5.95 (2.13) | 0.163 |  |
| CAD | 29 (9.6) | 7 (10.3) | 0.022 | 6 (10.0) | 6 (10.0) | <0.001 |  |
| HTN | 111 (36.9) | 34 (50.0) | **0.267** | 26 (43.3) | 28 (46.7) | 0.067 |  |
| CHF | 8 (2.7) | 0 (0.0) | **0.234** | 0 (0.0) | 0 (0.0) | <0.001 |  |
| CKD | 25 (8.3) | 6 (8.8) | 0.019 | 1 (1.7) | 3 (5.0) | 0.187 |  |
| DM | 66 (21.9 | 21 (30.9) | **0.204** | 18 (30.0) | 17 (28.3) | 0.037 |  |
| HLD | 76 (25.2) | 25 (36.8) | **0.251** | 18 (30.0) | 20 (33.3) | 0.072 |  |
| Obesity | 26 (8.6) | 6 (8.8) | 0.007 | 6 (10.0) | 4 (6.7) | 0.121 |  |
| Vaccinations | 111 (36.9) | 21 (30.9) | 0.127 | 17 (28.3) | 19 (31.7) | 0.073 |  |
| Creatinine rise | 255 (84.7) | 64 (94.1) | **0.309** | 55 (91.7) | 57 (95.0) | 0.134 |  |
| IMV | 301 (100.0) | 68 (100.0) | <0.001 | 60 (100.0) | 60 (100.0) | <0.001 |  |
| ECMO | 7 (2.3) | 11 (16.2) | **0.492** | 2 (3.3) | 11 (18.3) | **0.497** |  |
| NIMV Days | 2.29 (2.76) | 2.11 (1.99) | 0.077 | 2.08 (2.01) | 2.20 (2.07) | 0.057 |  |
| HFNC Duration | 3.83 (4.26) | 11.30 (13.89) | **0.727** | 5.44 (4.08) | 11.94 (14.59) | **0.607** |  |
| IMV Days | 9.40 (8.28) | 15.48 (15.09) | **0.499** | 10.83 (10.02) | 16.60 (15.54) | **0.442** |  |
| Vent free days | 12.18 (11.15) | 7.95 (9.68) | **0.405** | 12.14 (10.89) | 8.47 (9.55) | **0.358** |  |
| ECMO Duration | 16.43 (18.24) | 41.45 (24.38) | **1.162** | 22.00 (26.87) | 41.45 (24.38) | **0.758** |  |
| CRRT Duration | 10.57 (9.36) | 10.31 (10.44) | 0.026 | 8.50 (3.69) | 10.87 (10.56) | **0.299** |  |
| ICU LOS | 13.69 (9.41) | 29.84 (22.95) | **0.921** | 16.31 (10.96) | 32.54 (23.27) | **0.892** |  |
| ICU free days | 15.07 (7.46) | 6.72 (8.23) | **1.063** | 13.80 (8.25) | 5.82 (7.19) | **1.030** |  |
| Hospital LOS | 21.72 (13.13) | 35.33 (29.77) | **0.592** | 26.35 (18.09) | 37.78 (30.56) | **0.455** |  |
| Mortality | 119 (39.5) | 27 (39.7) | 0.003 | 26 (43.3) | 21 (35.0) | 0.171 |  |

SMD>0.2 signifies a significant difference between the groups

CAD: Coronary Artery Disease; HTN: Hypertension; CHF: Congestive Heart Failure; CKD: Chronic Kidney Disease; BMI: Body Mass Index, SOFA: Sequential Organ Failure Assessment; IMV: Invasive Mechanical Ventilation; NIV: Non-Invasive Ventilation; HFNC: High-Flow Nasal Cannula; ECMO: Extracorporeal Membrane Oxygenation; CRRT: Continuous Renal Replacement Therapy; LOS: Length of Stay

Supplementary Table 2: comparison among HFNC +/- iNO treated patients who underwent early intubation(<5days)

|  | Before Matching | | | After Matching | | |
| --- | --- | --- | --- | --- | --- | --- |
| Variables | No iNO used (n=236) | iNO used (n=31) | SMD | No iNO used (n=42) | iNO used (n=21) | SMD |
| Age | 61.21 (14.88) | 63.52 (12.83) | 0.166 | 64.45 (12.34) | 63.33 (13.44) | 0.087 |
| Female | 77 (32.6) | 11 (35.5) | 0.060 | 13 (31.0) | 6 (28.6) | 0.052 |
| Race |  | | 0.724 |  | | 0.170 |
| Caucasian | 202 (85.6) | 19(61.3) |  | 33 (78.6) | 16 (76.2) |  |
| American Indian/Alaska Native/ Pacific Islander | 4 (1.7) | 2 (6.5) |  | 0 (0.0) | 0 (0.0) |  |
| African American | 2 (0.8) | 4 (12.9) |  | 1 (2.4) | 1 (4.8) |  |
| Asian | 8 (3.4) | 4(12.9) |  | 5 (11.9) | 2 (9.5) |  |
| Mixed/Other/Unknown | 20 (8.5) | 2 (6.5) |  | 3 (7.1) | 2 (9.5) |  |
| BMI | 34.59 (8.87) | 31.12 (5.35) | 0.473 | 30.96 (5.43) | 31.39 (6.32) | 0.073 |
| NIV to IMV days | 1.72 (1.51) | 2.94 (1.53) | 0.796 | 1.57 (1.47) | 2.95 (1.69) | **0.873** |
| Highest SOFA | 12.23 (2.61) | 12.32 (2.80) | 0.032 | 12.55 (2.61) | 12.71 (2.28) | 0.068 |
| CAD | 24 (10.2) | 3 (9.7) | 0.016 | 3 (7.1) | 2 (9.5) | 0.086 |
| HTN | 87 (36.9) | 16 (51.6) | 0.300 | 19 (45.2) | 9 (42.9) | 0.048 |
| CHF | 7 (3.0) | 0 (0.0) | 0.247 | 0 (0.0) | 0 (0.0) | <0.001 |
| CKD | 17 (7.2) | 3 (9.7) | 0.089 | 1 (2.4) | 1 (4.8) | 0.129 |
| DM | 50 (21.2) | 10 (32.3) | 0.252 | 11 (26.2) | 7 (33.3) | 0.157 |
| HLD | 58 (24.6) | 14 (45.2) | 0.442 | 15 (35.7) | 9 (42.9) | 0.147 |
| Obesity | 20 (8.5) | 4 (12.9) | 0.144 | 4 (9.5) | 2 (9.5) | <0.001 |
| Vaccinations | 91 (38.6) | 10 (32.3) | 0.132 | 14 (33.3) | 6 (28.6) | 0.103 |
| Creatinine rise | 195 (82.6) | 29 (93.5) | 0.342 | 34 (81.0) | 21 (100.0) | **0.686** |
| IMV | 236 (100.0) | 31 (100.0) | <0.001 | 42 (100.0) | 21 (100.0) | <0.001 |
| ECMO | 2 (0.8) | 5 (16.1) | 0.570 | 1 (2.4) | 3 (14.3) | **0.441** |
| NIMV Days | 1.78 (1.80) | 1.53 (1.19) | 0.163 | 1.27 (1.20) | 1.83 (1.30) | **0.451** |
| HFNC Duration | 2.85 (3.42) | 6.47 (11.70) | 0.420 | 2.74 (3.07) | 4.68 (4.74 | **0.488** |
| IMV Days | 9.35 (7.70) | 15.82 (14.67) | 0.552 | 10.06 (7.88) | 16.01 (10.02) | 0.661 |
| Vent free days | 13.47 (10.77) | 9.32 (9.68) | **0.406** | 10.01 (10.56) | 8.55 (8.52) | 0.152 |
| ECMO Duration | 12.00 (7.07) | 42.40 (23.94) | 1.722 | 7.00 (NA) | 28.00 (15.00) | N/A |
| CRRT Duration | 10.74 (8.58) | 10.29 (12.27) | 0.043 | 17.00 (11.47) | 11.67 (12.83) | **0.438** |
| ICU LOS | 13.04 (8.32) | 24.51 (22.12) | 0.687 | 13.72 (9.09) | 23.58 (11.28) | **0.962** |
| ICU free days | 15.39 (7.04) | 9.88 (9.32) | **0.667** | 14.43 (7.54) | 7.20 (7.94) | **0.934** |
| Hospital LOS | 20.91 (13.24) | 28.45 (25.95) | 0.366 | 21.81 (14.08) | 27.19 (15.27) | **0.366** |
| Mortality | 77 (32.6) | 9 (29.0) | 0.078 | 18(42.9) | 6 (28.6) | **0.302** |

SMD>0.2 signifies a significant difference between the groups

CAD: Coronary Artery Disease; HTN: Hypertension; CHF: Congestive Heart Failure; CKD: Chronic Kidney Disease; BMI: Body Mass Index, SOFA: Sequential Organ Failure Assessment; IMV: Invasive Mechanical Ventilation; NIV: Non-Invasive Ventilation; HFNC: High-Flow Nasal Cannula; ECMO: Extracorporeal Membrane Oxygenation; CRRT: Continuous Renal Replacement Therapy; LOS: Length of Stay

Supplementary Table 3: Comparison between Responder versus non-responders- spontaneously breathing and post-intubation iNO

|  | Spontaneously breathing patients | | | Intubated | | |
| --- | --- | --- | --- | --- | --- | --- |
| Variables | Non-responder (n=52) | Responder (n=78) | SMD | Non responder (n=33) | Responder (n=151) | SMD |
| Age | 62.06 (13.28) | 61.79 (13.86) | 0.019 | 57.24 (14.23) | 56.84 (14.21) | 0.028 |
| Female | 17 (32.7) | 24 (30.8) | 0.041 | 10 (30.3) | 54 ( 35.8) | 0.116 |
| Race |  | | 0.188 |  | | **0.202** |
| Caucasian | 42 (80.8) | 61 (78.2) |  | 24 ( 72.7) | 112 ( 74.2) |  |
| American Indian/Alaska Native/ Pacific Islander | 1 ( 1.9) | 3 ( 3.8) |  | 2 ( 6.1) | 15 ( 9.9) |  |
| African American | 3 ( 5.8) | 7 (9.0) |  | 2 ( 6.1) | 7 ( 4.6) |  |
| Asian | 4 ( 7.7) | 5 (6.4) |  | 1 ( 3.0) | 5 ( 3.3) |  |
| Mixed/Other/ Unknown | 2 ( 3.8) | 2 (2.6) |  | 4 ( 12.1) | 12 ( 7.9) |  |
| BMI | 31.07 (5.92) | 31.17 (6.03) | 0.017 | 33.46 (4.71) | 33.17 (8.29) | 0.044 |
| Highest SOFA | 9.73 (4.45) | 7.78 (3.94) | 0.465 | 14.85 (2.13) | 13.66 (2.56) | **0.506** |
| CAD | 5 (9.6) | 7 ( 9.0) | 0.022 | 3 (9.1) | 9 (6.0) | 0.119 |
| HTN | 19 (36.5) | 34 (43.6) | 0.144 | 7 (21.2) | 56 (37.1) | **0.355** |
| CHF | 0 (0.0) | 1 (1.3) | 0.161 | 0 (0.0) | 2 (1.3) | 0.164 |
| CKD | 3 (5.8) | 6 (7.7) | 0.077 | 2 (6.1) | 12 (7.9) | 0.074 |
| DM | 10 (19.2) | 18 (23.1) | 0.094 | 2 (6.1) | 31 (20.5) | **0.436** |
| HLD | 16 (30.8) | 28 (35.9) | 0.109 | 4 (12.1) | 33 (21.9) | **0.261** |
| Obesity | 5 (9.6) | 9 (11.5) | 0.063 | 6 (18.2) | 17 (11.3) | 0.196 |
| Vaccinations | 17 (32.7) | 37 (47.4) | **0.304** | 10 (30.3) | 50 (33.1) | 0.060 |
| Creatinine rise | 41 (78.8) | 54 (69.2) | **0.221** | 31 (93.9) | 143 (94.7) | 0.033 |
| Intubated, % | 25 (48.19) | 20 (25.6) | **0.478** | 33 (100.0) | 151 (100.0) | <0.001 |
| NIMV Days | 2.26 (2.64) | 2.82 (2.92) | 0.201 | 1.93 (2.80) | 1.62 (1.97) | 0.128 |
| HFNC Duration | 12.39 (14.43) | 10.97 (9.12) | 0.118 | 7.92 (11.48) | 4.62 (6.42) | **0.355** |
| IMV days | 15.10 (13.83) | 20.88 (19.28) | **0.345** | 29.03 (28.31) | 22.83 (22.54) | **0.242** |
| Vent free days | 8.06 (10.26) | 4.20 (6.63) | **0.447** | 2.18 (5.92) | 5.98 (9.09) | **0.495** |
| ECMO Duration | 51.75 (27.81) | 31.50 (18.17) | **0.862** | 62.50 (31.16) | 39.76 (27.70) | **0.772** |
| CRRT Duration | 10.43 (10.67) | 11.80 (12.91) | 0.116 | 15.53 (16.42) | 15.65 (15.67) | 0.008 |
| ICU LOS | 21.43 (22.27) | 16.74 (16.86) | **0.237** | 35.97 (33.59) | 27.43 (26.29) | **0.283** |
| ICU free days | 11.73 (10.09) | 14.48 (9.54) | **0.281** | 0.93 (3.54) | 4.70 (7.56) | **0.638** |
| Hospital LOS | 28.98 (26.34) | 24.23 (21.87) | 0.196 | 40.68 (35.41) | 35.32 (33.32) | 0.156 |
| Mortality | 15 (28.8) | 14 (17.9) | **0.260** | 24 (72.7) | 68 (45.0) | **0.587** |
| Nitric Oxide Starting Dose | 19.97 (7.74) | 21.82 (8.24) | **0.232** | 20.95 (8.72) | 25.00 (9.62) | **0.440** |
| Nitric Oxide Max dose | 30.08 (13.97) | 25.38 (9.94) | **0.387** | 25.52 (14.19) | 28.72 (12.11) | **0.243** |
| Respiratory rate just before iNO initiation | 23.52 (6.49) | 24.96 (7.12) | **0.212** | 22.53 (6.38) | 23.57 (8.78) | 0.135 |
| FiO2 just before iNO initiation | 0.87 (0.23) | 0.92 (0.13) | **0.280** | 0.76 (0.22) | 0.87 (0.18) | **0.497** |
| PaO2 just before iNO initiation | 59.35 (13.74) | 72.60 (25.89) | **0.639** | 90.53 (31.89) | 76.64 (29.33) | **0.453** |
| P/F ratio pre-iNO | 76.21 (51.74) | 85.19 (59.54) | **0.161** | 158.64 (123.11) | 91.32 (48.58) | **0.719** |

SMD>0.2 signifies significant difference between the groups

CAD: Coronary Artery Disease; HTN: Hypertension; CHF: Congestive Heart Failure; CKD: Chronic Kidney Disease; BMI: Body Mass Index, SOFA: Sequential Organ Failure Assessment; IMV: Invasive Mechanical Ventilation; NIV: Non-Invasive Ventilation; HFNC: High-Flow Nasal Cannula; ECMO: Extracorporeal Membrane Oxygenation; CRRT: Continuous Renal Replacement Therapy; LOS: Length of Stay

Supplementary Table 4: Comparison between intubated versus non-intubated groups among those who received iNO while on HFNC

| Variables | Total (N=153) | Intubated  (N=68) | Non-intubated  (N=85) | SMD |
| --- | --- | --- | --- | --- |
| Age | 62.03 (13.47) | 63.43 (11.94) | 60.92 (14.55) | 0.188 |
| Female Sex | 52 (34.0%) | 28 (41.2) | 24 (28.2) | 0.274 |
| Race | | | | **0.351** |
| Caucasian | 119 (77.8%) | 49 (72.1) | 70 (82.4) |  |
| American Indian/Alaska Native/ Pacific Islander | 5 (3.3%) | 4 (5.9) | 1 (1.2) |  |
| African American | 12 (7.8%) | 5 (7.4) | 7 (8.2) |  |
| Asian | 11(7.2%) | 6 (8.8) | 5 (5.9) |  |
| Mixed/Other/Unknown | 6 (3.9%) | 4 (5.9) | 2 (2.4) |  |
| BMI | 31.30 (6.07) | 30.93 (5.85) | 31.59 (6.25) | 0.110 |
| Highest SOFA | 9.02 (4.24) | 12.62 (2.68) | 5.86 (2.47) | **2.627** |
| Baseline SOFA | 5.12 (2.08) | 5.98 (2.17) | 4.36 (1.68) | **0.839** |
| CAD | 15 (9.8%) | 7 (10.3) | 8 (9.4) | 0.030 |
| HTN | 66 (43.1%) | 34 (50.0) | 32 (37.6) | **0.251** |
| CHF | 1 (0.7%) | 0 (0.0) | 1 (1.2) | 0.154 |
| CKD | 11 (7.2%) | 6 (8.8) | 5 (5.9) | 0.113 |
| DM | 37 (24.2%) | 21 (30.9) | 16 (18.8) | 0.282 |
| HLD | 55 (35.9%) | 25 (36.8) | 30 (35.3) | 0.031 |
| Pre-Hospital Obesity Diagnosis | 16 (10.5%) | 6 (8.8) | 5 (5.9) | 0.113 |
| COVID-19 Vaccination | 64 (41.8%) | 21 (30.9) | 43 (50.6) | **0.409** |
| Baseline code status  Full Code  No CPR, Yes Intubation  Yes CPR, No Intubation  DNR/DNI | 143 (93.5)  1 (0.7)  1 (0.7)  8 (5.2)  0 (0.0) | 64 (94.1)  1 (1.5)  1 (1.5)  2 (2.9) | 79 (92.9)  0 (0.0)  0 (0.0)  6 (7.1) | **0.308** |
| Creatinine rise | 115 (75.2%) | 64 (94.1) | 51 (60.0) | **0.888** |
| ECMO | 13 (8.5%) | 11 (16.2) | 2 (2.4) | **0.491** |
| CRRT | 16 (10.5%) | 16 (23.5) | 0 (0.0) | **0.784** |
| HFNC Duration (mean) | 11.03 (11.95) | 11.30 (13.89) | 10.82 (10.24) | 0.039 |
| NIMV Days (mean) | 2.34 (2.61) | 2.11 (1.99) | 2.60 (3.20) | 0.187 |
| ECMO Duration Hospitalization (mean) | 38.62 (24.98) | 41.45 (24.38) | 23.00 (31.11) | **0.660** |
| CRRT duration (mean) | 10.31 (10.44) | 10.31 (10.44) | - | - |
| ICU LOS (mean) | 18.82 (19.42) | 29.84 (22.95) | 9.81 (8.82) | **1.151** |
| ICU-free days | 13.23 (9.68) | 6.72 (8.23) | 18.62 (7.16) | **1.543** |
| Hospital LOS (mean) | 26.05 (23.96) | 35.33 (29.77) | 18.62 (14.38) | **0.715** |
| In-hospital mortality | 36 (23.5%) | 27 (39.7) | 9 (10.6) | **0.712** |

SMD>0.2 signifies significant difference between the groups

CAD: Coronary Artery Disease; HTN: Hypertension; CHF: Congestive Heart Failure; CKD: Chronic Kidney Disease; BMI: Body Mass Index, SOFA: Sequential Organ Failure Assessment; IMV: Invasive Mechanical Ventilation; NIV: Non-Invasive Ventilation; HFNC: High-Flow Nasal Cannula; ECMO: Extracorporeal Membrane Oxygenation; CRRT: Continuous Renal Replacement Therapy; LOS: Length of Stay
